# Targeted volumetric single-molecule localization microscopy of defined presynaptic structures in brain sections

**DOI:** 10.1038/s42003-021-01939-z

**Published:** 2021-03-25

**Authors:** Martin Pauli, Mila M. Paul, Sven Proppert, Achmed Mrestani, Marzieh Sharifi, Felix Repp, Lydia Kürzinger, Philip Kollmannsberger, Markus Sauer, Manfred Heckmann, Anna-Leena Sirén

**Affiliations:** 1 Department for Neurophysiology, Institute for Physiology, Julius-Maximilians-University Würzburg, Würzburg, Germany; 2 Department of Neurosurgery, University Hospital of Würzburg, Würzburg, Germany; 3 Center for Computational and Theoretical Biology, Julius-Maximilians-University Würzburg, Würzburg, Germany; 4 Department of Biotechnology and Biophysics, Biocenter, Julius-Maximilians-University Würzburg, Würzburg, Germany

**Keywords:** Synaptic vesicle exocytosis, Fluorescence imaging

## Abstract

Revealing the molecular organization of anatomically precisely defined brain regions is necessary for refined understanding of synaptic plasticity. Although three-dimensional (3D) single-molecule localization microscopy can provide the required resolution, imaging more than a few micrometers deep into tissue remains challenging. To quantify presynaptic active zones (AZ) of entire, large, conditional detonator hippocampal mossy fiber (MF) boutons with diameters as large as 10 µm, we developed a method for targeted volumetric direct stochastic optical reconstruction microscopy (*d*STORM). An optimized protocol for fast repeated axial scanning and efficient sequential labeling of the AZ scaffold Bassoon and membrane bound GFP with Alexa Fluor 647 enabled 3D-*d*STORM imaging of 25 µm thick mouse brain sections and assignment of AZs to specific neuronal substructures. Quantitative data analysis revealed large differences in Bassoon cluster size and density for distinct hippocampal regions with largest clusters in MF boutons.

## Abstract (web summary)

Pauli et al. develop targeted volumetric *d*STORM in order to image large hippocampal mossy fiber boutons (MFBs) in brain slices. They can identify synaptic targets of individual MFBs and measured size and density of Bassoon clusters within individual untruncated MFBs at nanoscopic resolution.

## Introduction

Synapses have been imaged in defined functional states with electron microscopy (EM)^1–4^. Serial sectioning^5^ and serial block-face^6^ scanning EM enabled 3D reconstructions of large volumes with high spatial resolution. On the other hand, proteins can be quantified in synapses using freeze fracture EM^3,7^. Biochemical analysis and super-resolution light microscopy of synaptosomes and hippocampal cultures have led to a 3D model of an “average” synapse^8^. However, since synapses differ substantially from one another even in defined structures, e.g., along the ventro-dorsal axis of the hippocampus (a key brain area for learning and memory^9^) quantitative information with nanometer spatial resolution in large tissue blocks is highly desirable.

So far single molecule localization microscopy (SMLM) was used successfully to study cultured hippocampal synapses^10–14^. In addition, 3D-SMLM imaging was achieved in brain sections using oil-immersion objectives to visualize the distribution of synaptic proteins in a plane a few micrometers above the coverslip^10,13^. More recently, self-interference 3D super-resolution microscopy and active point-spread function (PSF) shaping in combination with adaptive optics were introduced to enable 3D localization of emitters in tissue with a thickness of up to 50 µm^15,16^. The latter approach allowed reconstructing super-resolution volumes with an axial depth of several micrometers. In cultured cells 4Pi single marker switching nanoscopy with deformable mirrors allowed imaging of individual cells up to 9 µm depth^17^. However, so far molecular imaging of well-defined larger regions of interest, e.g., all active zones (AZs) in an entire mossy fiber (MF) bouton was not achieved. Imaging entire boutons is highly desirable to further clarify plasticity between individual boutons and would nicely complement recent progress using other techniques^18,19^. Both, photobleaching of fluorophores and inefficient labeling of target proteins due to the restricted penetration of antibodies render quantitative molecular imaging, an intrinsic strength of SMLM^14,20^, in thick tissue samples complicated.

**Fig. 1:**
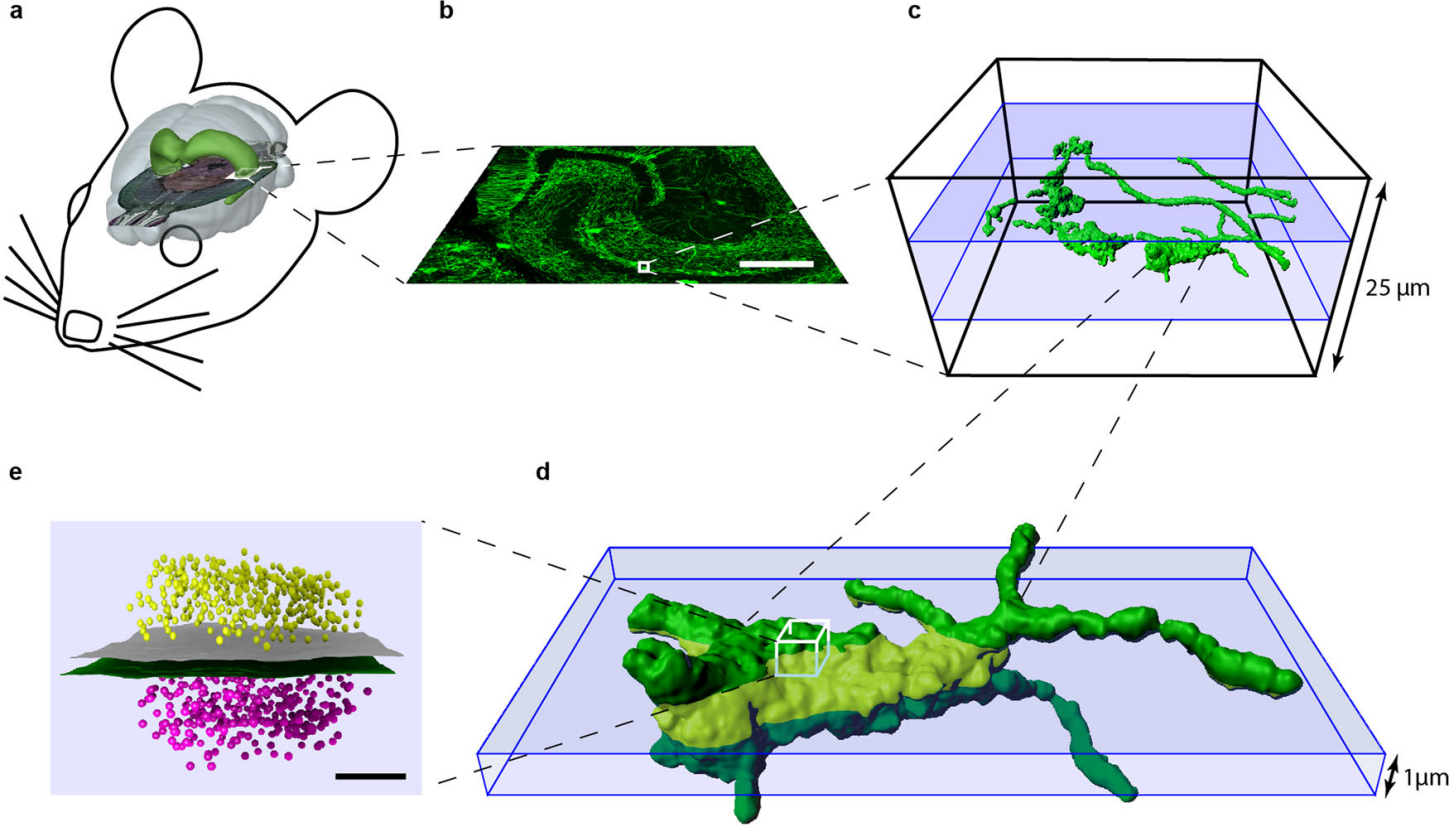
Schematic overview for targeted imaging of hippocampal mossy fiber synaptic contacts. **a** View of a virtual mouse brain with the dimensions of the hippocampal formation in green and a horizontal section from Allen Mouse Brain Atlas (image credit: Allen Institute for Brain Science, available from: http://mouse.brain-map.org/static/brainexplorer) to demonstrate the hippocampal region used for imaging (white box). **b** Confocal image of mossy fibers in a 25 µm thick hippocampal slice from a Thy1mEGFP(Lsi1) mouse. The white box marks again the region of interest (25 µm × 25 µm × 25 µm) that is shown magnified in the next panel. **c** 3D volume reconstruction of mEGFP in *xyz*-view, showing axons and mossy fiber boutons (green), a typical bouton with filopodial extensions is marked and further highlighted in **d**. The window (10 µm in *z*) for *en bloc* imaging in the 25 µm thick tissue block is shown in blue. **d** 3D volume reconstruction of a mossy fiber bouton. The light green area of the bouton shows the volume that would be captured using a typical 1 µm imaging depth in *z* for a single focal plane. The white box marks a virtual synaptic contact that is further magnified in the next panel. **e** A virtual synaptic contact in side view (presynaptic membrane in green, Bassoon clusters in magenta, Homer-1 in yellow, postsynaptic membrane in gray), scale bar in **b** 200 µm, in **e** 100 nm.

We focus on large hippocampal mossy fibers boutons (MFBs)^21–23^ in brain slices (Fig. 1). These so-called conditional detonators are complex structures with large diameters and tentacle like filopodial extensions^5–7^. A single MFB may contain 18–45 separate but very closely spaced active zones (AZs) harboring transmitter release sites^6,24^, and up to 25,000 synaptic vesicles with 1400–5700 vesicles as a readily releasable pool^5,25^. MFBs show remarkable presynaptic plasticity and high variability in presynaptic patch clamp and capacitance measurements^25,26^, a low release probability (0.01–0.05)^27^ and an estimated coupling distance of 75 nm^26^. Neither bouton size nor peak calcium current amplitude predict release^25^, therefore it is likely that the molecular organization of AZs controls plasticity of MFB AZs^21,22,26,28^. Importantly, synaptic contacts formed by hippocampal mossy fibers (MFs) display target-specific information processing and plasticity^21,22^. While the glutamatergic synaptic contacts between large MFBs and cornu ammonis (CA)3 pyramidal cells are excitatory and rely on presynaptic forms of plasticity, the contacts between filopodial extensions^29^ and interneurons may be inhibitory and depend both on presynaptic and postsynaptic mechanisms to induce long term-depression^23,24,27,30^. Thus, imaging of plasticity at these contacts necessitates nanoscopic resolution and precise anatomical identification of the synaptic contacts.

We developed targeted volumetric direct stochastic optical reconstruction microscopy (*d*STORM) to measure size and density of Bassoon^28,31^ clusters as markers for presynaptic AZs in large MFBs. In view of plasticity we expected variability between individual animals, along the ventro-dorsal axis of the hippocampus and between the different synaptic targets of MFBs. We started with 2D-imaging in 1 µm thin sections. This allowed to compare synaptic contacts in defined regions and between individual animals. The data were, however, limited by potentially overlapping projections and mechanical truncation. 3D-scanning of large volumes in 25 µm thick sections reduced projection artifacts and truncation of Bassoon clusters, and provided large samples within single tissue sections of individual animals. This uncovered significant differences for neighboring hippocampal regions within one tissue section, but did not provide clear evidence for Forskolin induced plasticity in the MFT. Different imaging approaches yielded similar dimensions of bassoon clusters indicating reliable measurements, thus leaving heterogeneity of the synaptic targets and their functional status as putative explanations for the observed variability of Forskolin induced changes. Finally, we show how sequential scanning allows to assign Bassoon clusters to identified synaptic targets of individual MFBs and measured size and density of Bassoon clusters within individual untruncated MFBs at nanoscopic resolution.

## Results

### Imaging synaptic contacts of hippocampal mossy fibers

Essential elements of the imaging workflow are illustrated in Fig. 1. It summarizes key steps for imaging active zones of entire large presynaptic elements with nanometer resolution in anatomically defined regions of the hippocampus. Crucial are: 1) targeted cutting, 2) optimal and homogeneous labeling, and 3) thick tissue blocks and 3D scanning to overcome truncation of synapses.

### Differences of Bassoon cluster size along ventro-dorsal axis of the hippocampus

**Fig. 2:**
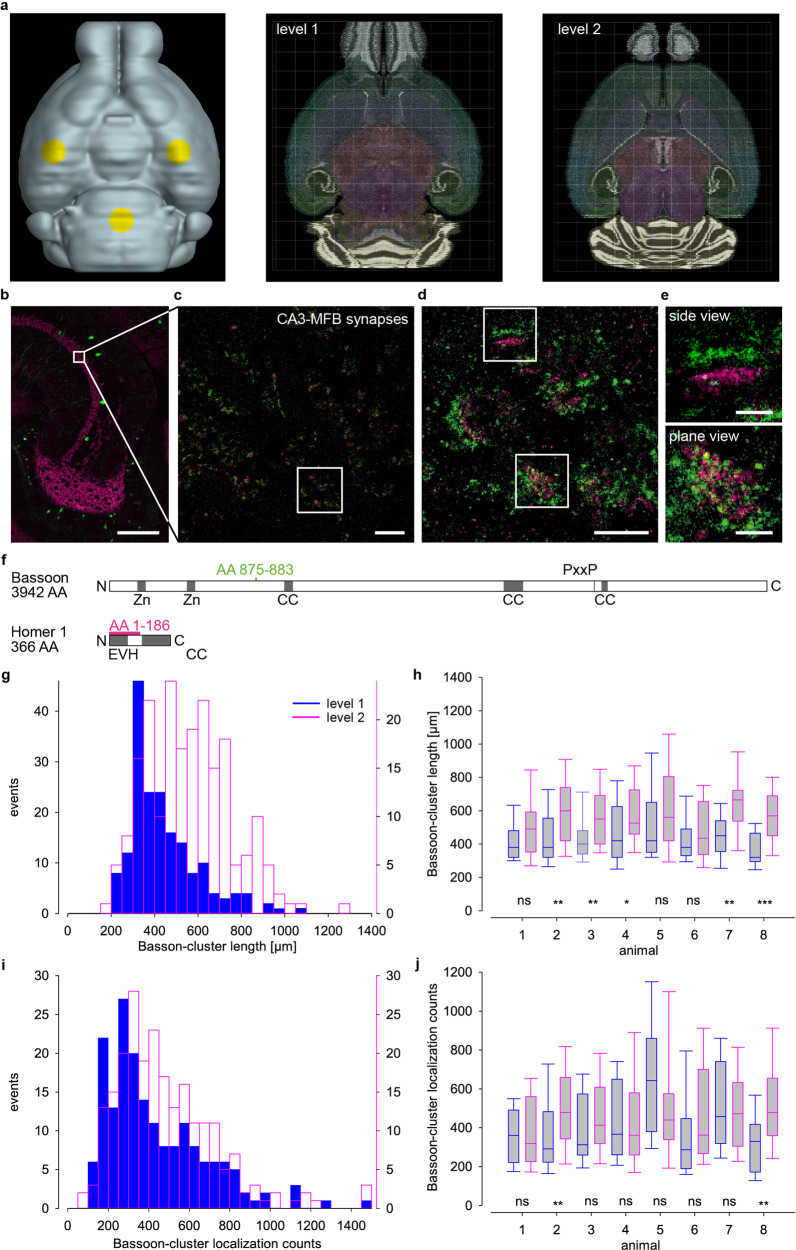
Two-color imaging of hippocampal mossy fibers and Bassoon clusters along the ventro-dorsal axis. **a** Ventral view of a virtual mouse brain with landmarks for trimming in yellow (left) and horizontal sections from Allen Mouse Brain Atlas (image credit: Allen Institute for Brain Science, available from: https://mouse.brain-map.org/static/brainexplorer) to illustrate the ventral (level 1, middle panel) and the 600 µm more dorsal region (level 2). **b** Confocal image of mossy fibers in a Thy1EGFP(M) mouse with zinc-transporter 3 (magenta) staining. A white box marks the imaging window (30 µm × 30 µm) shown enlarged in the next panel. **c** Two-color *d*STORM image of synapses stained for presynaptic Bassoon (green) and postsynaptic Homer 1 (magenta). White box indicates magnified view in the next panel. **d**, **e** Synaptic contacts in different orientations: upper box—side view, lower box—plane view further magnified in **e**. **f** Protein domain layout of *Mus musculus* Bassoon and Homer1: Zinc-finger (Zn), coiled coil (CC), PxxP, and enabled/VASP homology (EVH); epitopes of the anti-Bassoon (green) and anti-Homer 1 (magenta) antibodies. Histograms of length (**g**) and counts (**i**) of Bassoon clusters in side view at level 1 (blue, *n* = 181 clusters in nine images from eight animals) and level 2 (magenta, *n* = 208 clusters in eight images from eight animals). Clusters at level 2 are longer (*p* < 0.001) and have more counts (*p* < 0.05) than at level 1. Summary box plots (horizontal line median, boxes 25th and 75th, whiskers 10th and 90th percentile) in all eight individual animals for length (**h**) and localization counts (**j**) (*p < 0.05; **p < 0.01; ***p < 0.001). **i** Scale bars 100 µm (**b**), 3 µm (**c**), 1 µm (**d**), and 200 nm (**e**).

Imaging in anatomically precisely defined regions demands standardized sectioning protocols. Acute 300 µm-thick slices are routinely used for electrophysiological recordings^3,25–27^, which gives a distance of 600 µm from the ventral to the dorsal surface of two successive slices. We decided for two anatomical levels of the hippocampus: A ventral level 1 and a dorsal level 2 which were 600 µm apart from each other (Fig. 2a). To precisely set imaging windows (30 µm × 30 µm) in the middle of the MF tract, we used Thy1EGFP(M) mice with strong cytoplasmic expression of EGFP in MFs^30^. The position of the imaging windows in MFs was further confirmed with anti-Zinc-transporter 3 (ZnT-3) staining (Fig. 2b). Thin 1 µm horizontal sections are ideal for single-molecule multicolor imaging using Alexa532 and Alexa647. We used two-color 2D-*d*STORM with antibodies directed against the presynaptic AZ protein Bassoon^31^, and the postsynaptic density (PSD) protein Homer 1^32^ (Fig. 2c–e). These are two well-characterized presynaptic and postsynaptic proteins, which are expressed in large mossy fiber boutons and CA3 pyramidal dendrites^7,31–33^. Bassoon contains multiple domains for interaction with other AZ components such as RIM-binding protein (RBP), Piccolo, and other proteins^31,34^. Its orientation within the AZ is unclear but it seems to be attached in membrane proximity via its PxxP-motif interaction with RBP (Fig. 2f). The designated epitope of the commercial monoclonal mouse antibody against Bassoon^10,31^ covers several hundred amino acids within a so-called Piccolo-Bassoon homology region^31^. To clarify Bassoon specificity epitope mapping was employed and identified a highly specific binding site of nine amino acids from position 875 to 883 (DTAVSGRGL) in the N-terminal region of Bassoon (Fig. 2f). As Homer 1 is a relatively small protein, known to form a mesh within the PSD^32^ with its C-terminal and N-terminal portions close to each other a precise epitope mapping of the commercial polyclonal antibody for Homer was not necessary (Fig. 2f).

We found many contacts in random orientations in each image (Fig. 2c, d) and focused on those in side views with parallel presynaptic Bassoon (green) and postsynaptic Homer 1 (magenta) (Fig. 2e, upper panel). Peak-to-peak distance of Bassoon and Homer 1 clusters was on average 149 ± 12 nm (mean ± SD) with little variability (Supplementary Fig. S1a–c). Bassoon clusters in side views were on average 376 ± 136 nm long, had a cross section width of 74 ± 16 nm and 139 ± 80 localization counts per cluster whereas clusters in plane view were 460 ± 155 nm long, had a width of 383 ± 98 nm and 297 ± 173 counts per cluster (Supplementary Fig. S1d–k). We imaged Bassoon clusters in side views of eight 24-week-old male Thy1-EGFP(M) mice (Fig. 2g–j). Bassoon cluster lengths and Bassoon localization counts were significantly different at the two levels (Fig. 2g, i), and similar differences were obtained when comparing individual animals (Fig. 2h, j). Since Bassoon cluster size and neurotransmitter release are correlated^35^, the gradient observed here between level 1 and 2 likely contributes to the high variability observed in recordings from MFBs^25–27^. Furthermore, these differences reinforce the requirement to precisely define regions of interest within mouse brains with submillimeter precision. In 2D imaging, as illustrated in Fig. 2g, h, some clusters were very large with a length reaching 1000 µm. Considering the complex, partially folded structure of hippocampal MFBs^6,24,29^ it is conceivable that in 2D imaging neighboring Bassoon clusters are superimposed, and thus appear larger than they actually are. The problem of overestimation decreases with smaller *z*-range but might even be relevant in 1 µm thin sections.

### Distinct Bassoon-cluster size in hippocampal sub compartments

**Fig. 3:**
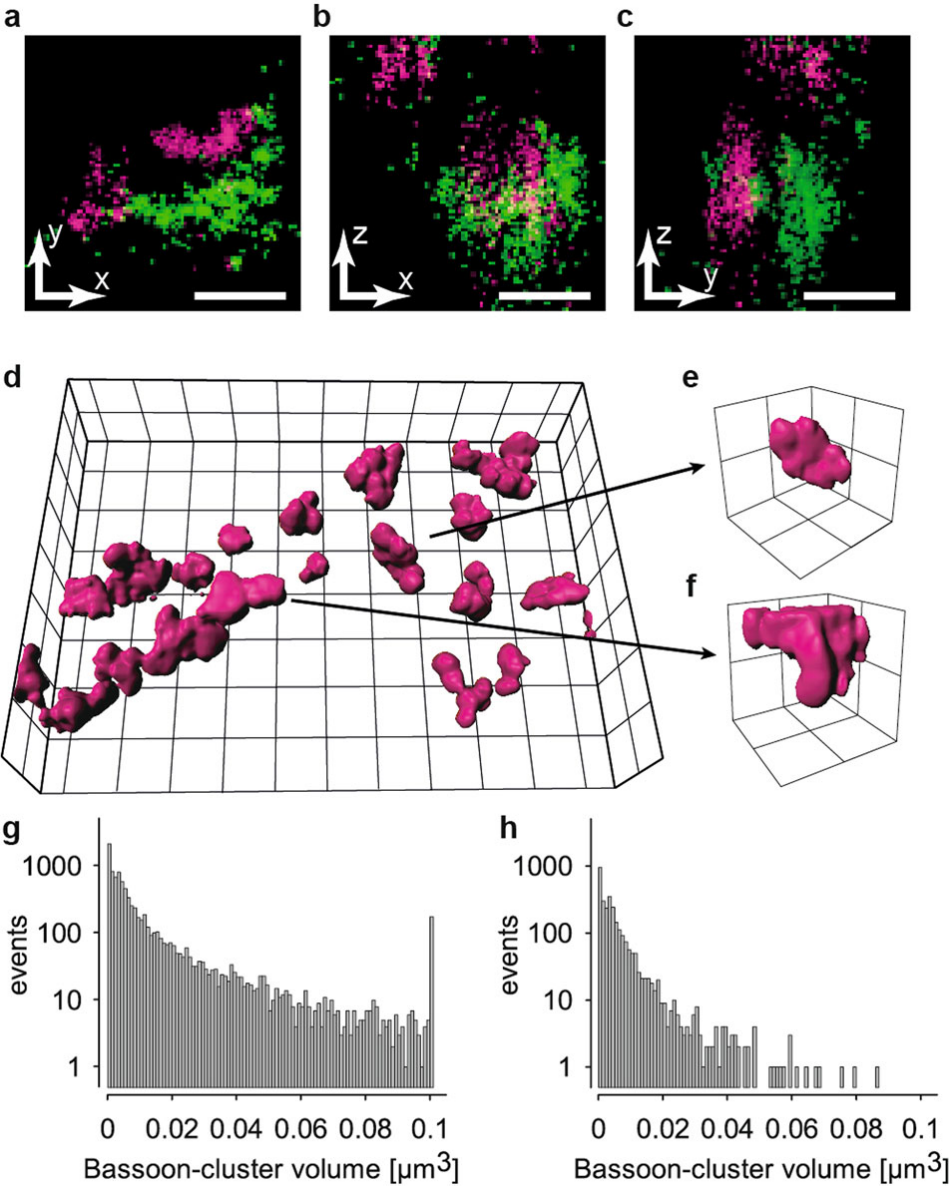
3D imaging uncovered truncation of Bassoon clusters. **a**–**c** Representative two-color 3D *d*STORM images of synaptic contacts with presynaptic Bassoon (green) and postsynaptic Homer 1 (magenta) in all three orientations (*xy*-view, *xz*-view, and *yz*-view; see Suppl. Video 1. **d** Volume reconstruction of a dataset of Bassoon. An entire cluster (**e**) within the imaged tissue volume, and a truncated cluster (**f**) extending beyond its upper edge are shown. Semi-logarithmic histograms of cluster volumes in seven images from 1 animal for all clusters (**g**, *n* = 8956) and for selected non-truncated clusters (**h**, *n* = 2925). Scale bars in **a**–**c** 100 nm, grid size in **d** 2 µm and in **e**, **f** 500 nm.

To minimize the chance that two clusters are misinterpreted as one in 2D projections, we implemented 3D-*d*STORM (Fig. 3). Figure 3a shows a Bassoon cluster in *xy*-projection appearing to extend to the left. Figure 3b, c show xz-projections and yz-projections and reveal two near-by clusters (Supplementary Video 1). 3D-imaging thus effectively solved the problem caused by overlapping 2D projections. A remaining limitation of these images is that many clusters extend beyond the borders of the 1 µm thin brain section, and are thus truncated as demonstrated (Fig. 3d–f) in an example of an entire cluster within the slice shown in Fig. 3e and a truncated one at the upper border of the brain slice (Fig. 3f) and in the histograms of the clusters in seven images from one animal (Fig. 3g, h). Re-evaluation of the data filtered for truncated clusters reduced cluster volume and variability (median ± 25th–75th percentile 0.0040 ± 0.0011–0.0113 µm^3^, *n* = 8956 in non-filtered data vs. median 0.0029 ± 0.0007–0.0058 µm^3^, *n* = 2925 in filtered data) (Fig. 3g, h). In the MFT imaging 1 µm thin sections thus lead to underestimation of cluster size.

**Fig. 4:**
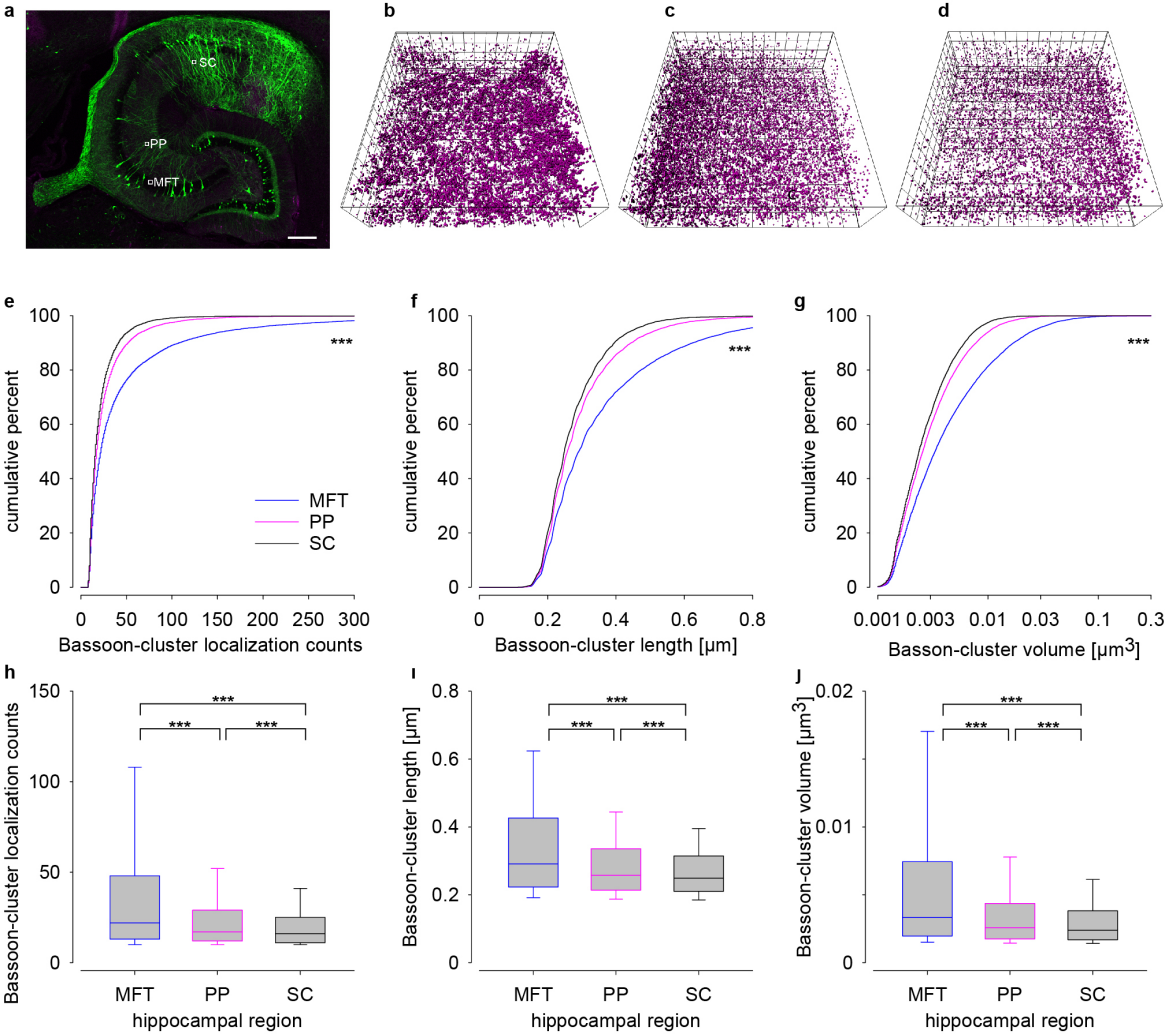
En bloc 3D imaging in 25 µm thick tissue slice revealed different Bassoon patterns in three distinct hippocampal circuits. **a** Confocal overview of a 25 µm thick section of the hippocampus in a Thy-1 EGFP(M) mouse with imaging windows in the mossy fiber tract (MFT), perforant pathway (PP), and Schaffer collaterals (SC) depicted to scale (25 × 25 µm). 3D plots of Bassoon clusters in MFT (**b**), PP (**c**), and SC (**d**). Cumulative plots of localization counts (**e**), cluster length (**f**), and volumes (**g**) in MFT (blue), PP (magenta), and SC (black). Corresponding data as box plots in **h**–**j** Scale bar: 200 µm in **a** and grid size 2 µm in **b**–**d**. Asterisks (***p < 0.001) denote statistical significance MFT vs. PP/SC and PP vs. SC.

To reduce truncation, we imaged larger tissue volumes en bloc in 25 µm sections using optimized procedures for homogenous labeling, Alexa 647 and a water immersion objective (see “Methods” section and Supplementary Figs. S2, S3, and S4). Alexa 532 could not be used in thick sections because of insufficient signal to noise ratios due to high background of short wavelength fluorophores^14,36^. We imaged three different regions (Fig. 4a), MF tract (MFT), perforant path (PP), and Schaffer collaterals (SC) in the same tissue slice in less than 3 h using Thy-1 EGFP fluorescence as a marker for hippocampal architecture. This enabled an unbiased evaluation of clusters with identical staining conditions for all regions without inter-animal variability. We observed different distributions of clusters in MFT (Fig. 4b), PP (Fig. 4c), and SC (Fig. 4d) with smallest and most sparse clusters in SC. Bassoon localization counts (median ± 25th–75th percentile: 22 ± 13–48), cluster length (0.291 ± 0.223–0.426 µm) and volume (0.0033 ± 0.0020–0.0075 µm^3^) in MFT were significantly larger (*p* < 0.001) compared to PP (17 ± 12–29; 0.257 ± 0.214–0.336 µm; 0.0026 ± 0.0018–0.0044 µm^3^) and SC (16 ± 11–25; 0.249 ± 0.210–0.315 µm; 0.0024 ± 0.0017–0.0038 µm^3^) (Fig. 4e–j). Bassoon cluster density in PP (6678 ± 6236–7194 counts/µm^3^) was significantly higher than in MFT (6428 ± 5959–7000 counts/µm^3^, *p* < 0.001) or in SC (6651 ± 6180–7207 counts/µm^3^, *p* < 0.01) (Supplementary Fig. S5a, b, e). In these experiments *n* = 11,232 for MFT, *n* = 12,596 for PP, and *n* = 8669 for SC.

### Effect of Forskolin

**Fig. 5:**
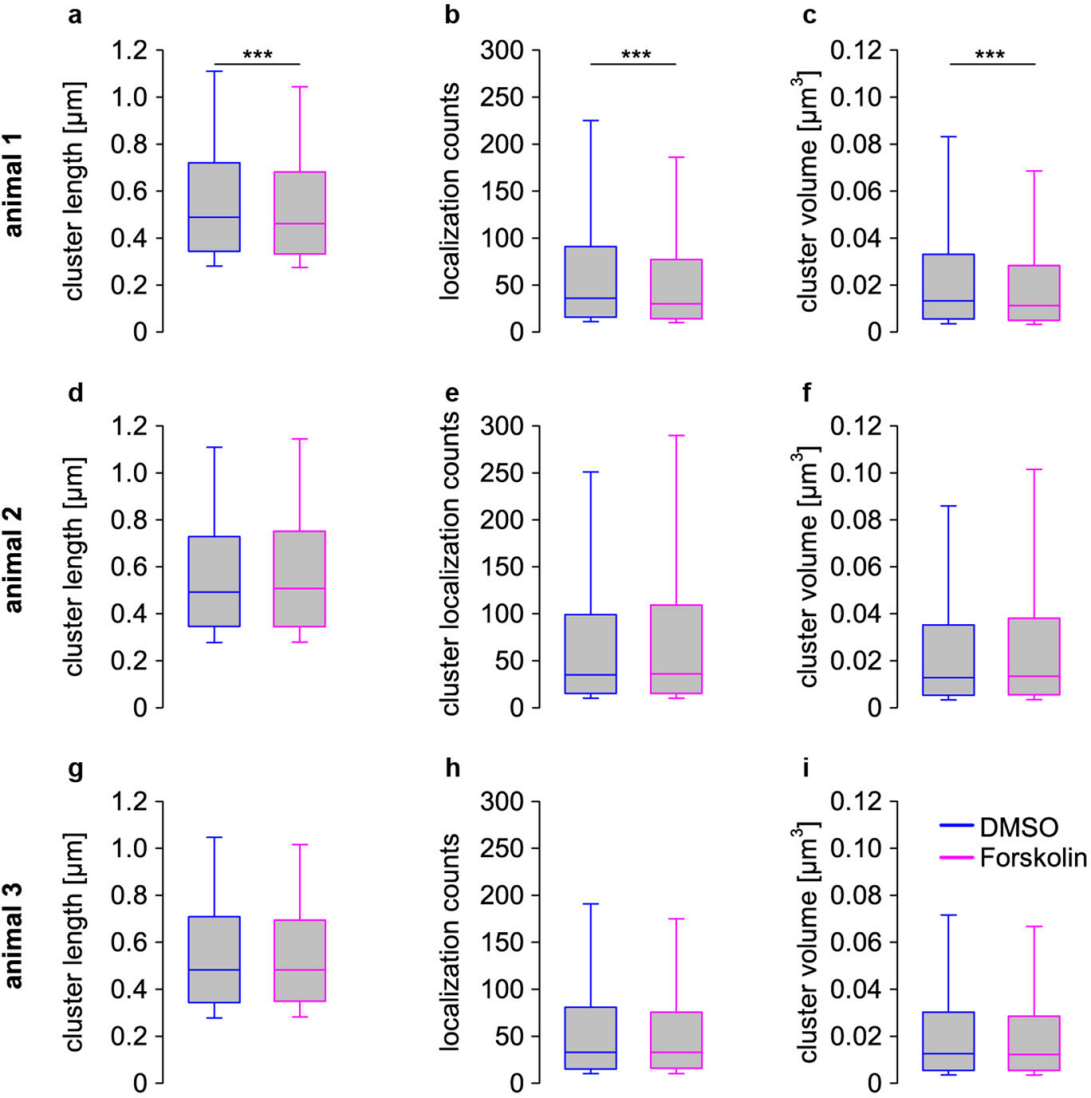
Forskolin induced plasticity. Bassoon cluster measures in scanning *d*STORM of 25 µm cryosections obtained from 300 µm acute brain sections of hippocampal mouse tissue; control group treated with DMSO (blue) experimental group treated with Forskolin solved in DMSO (magenta). One out of three animals showed highly significant changes (*p* < 0.001) in **a** Bassoon-cluster length (median ± 25th–75th percentile; DMSO: 0.490 ± 0.344–0.721 µm; Forskolin: 0.462 ± 0.333–0.681 µm) **b** localization counts (DMSO: 36 ± 16–91; Forskolin: 30 ± 14–77) and **c** volume (DMSO: 0.0132 ± 0.0056–0.0331 µm^3^; Forskolin: 0.0112 ± 0.0050–0.0283 µm^3^); Animal 2: **d** length (DMSO: 0.482 ± 0.343–0.709 µm; Forskolin: 0.482 ± 0.349–0.694 µm), **e** counts (DMSO: 33 ± 15–81; Forskolin: 33 ± 16–75.5), **f** volume (DMSO: 0.0125 ± 0.00544–0.0302 µm^3^; Forskolin: 0.0123 ± 0.00548–0.0285 µm^3^); Animal 3: **g** length (DMSO: 0.493 ± 0.345–0.729 µm; Forskolin: 0.507 ± 0.345–0.752 µm), **h** counts (DMSO: 35 ± 15–99; Forskolin: 36 ± 15–109), **i** volume (DMSO: 0.0127 ± 0.00526–0.0352 µm^3^; Forskolin: 0.0134 ± 0.00541–0.0381 µm^3^).

The ability to obtain large samples led us to address if it would be possible to capture Forskolin induced plasticity in MFT^18,37^. Thus, we measured Bassoon clusters in MFT using scanning *d*STORM in 25 µm cryosections obtained from 300 µm acute brain sections after Forskolin. In one out of three animals, we found highly significant decreases (*p* < 0.001) in Bassoon cluster length (median ± 25th–75th percentile; DMSO: 0.490 ± 0.344–0.721 µm; Forskolin: 0.462 ± 0.333–0.681 µm); localization counts (DMSO: 36 ± 16–91; Forskolin: 30 ± 14–77) and cluster volume (DMSO: 0.0132 ± 0.0056–0.0331 µm^3^; Forskolin: 0.0112 ± 0.0050–0.0283 µm^3^). However, no significant differences in these parameters were found in two other animals (Fig. 5a–c) or in the pooled Bassoon cluster density (Supplementary Fig. S5c, f). Control values were similar in all three animals. Recent work on MFBs in slice cultures interestingly reported also no massive changes in AZ number or size after Forskolin treatment using stimulated emission depletion (STED) microscopy^18^. Furthermore, the same authors reported mean AZ length of 0.34 ± 0.2 µm for DMSO and 0.31 ± 0.2 µm for Forskolin, not significantly different using electron tomography (Maus et al.^18^, Supplementary Table 1).

### Quantitative reliability of Bassoon cluster measures

**Fig. 6:**
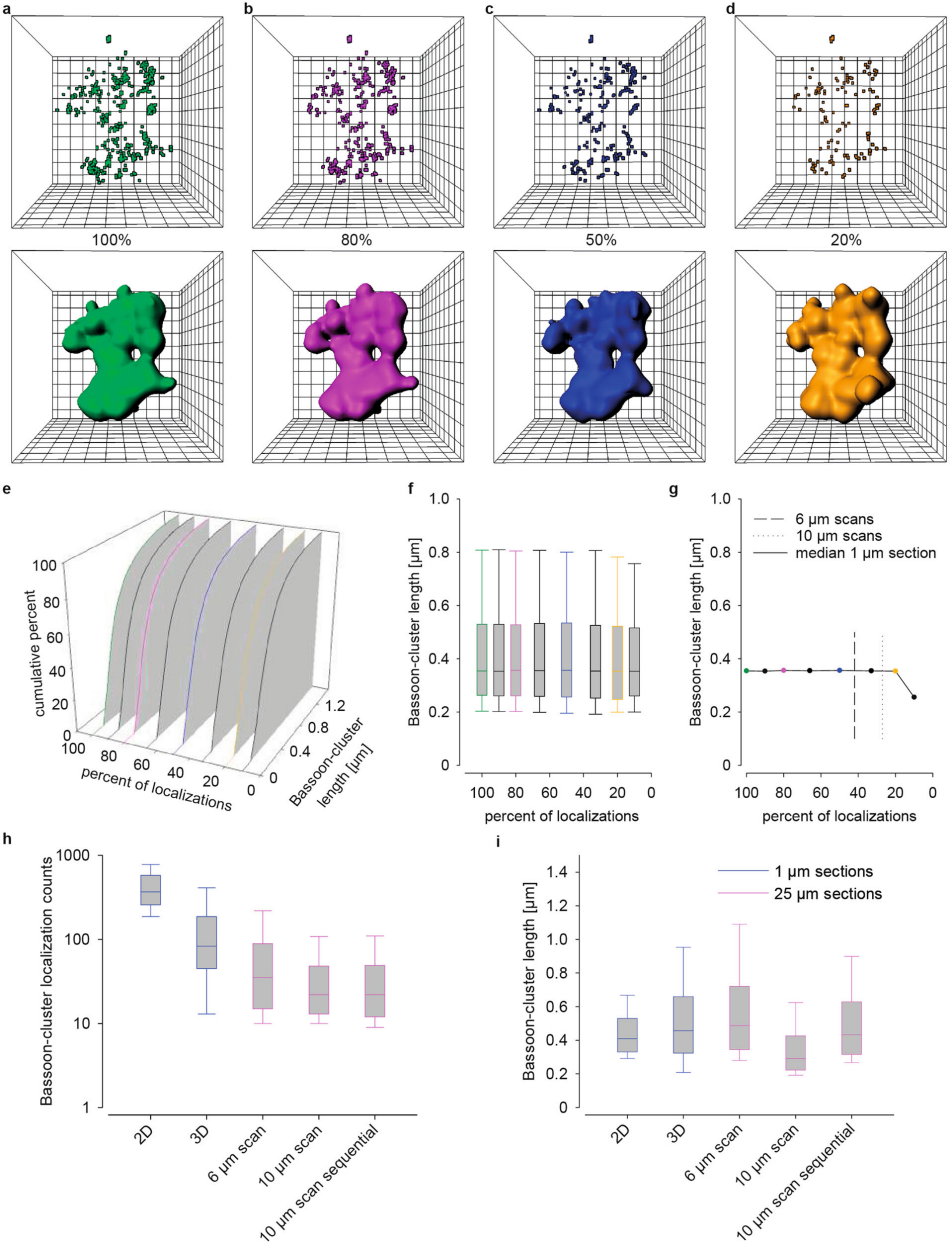
Comparison of Bassoon cluster counts and length in the mossy fiber tract. **a**–**d** Bassoon-cluster localizations (upper images) and their 3D volume reconstruction in a 1 µm scan and in artificial low-density data sets created by reducing counts to 80, 50, and 20% of the original counts. Mesh width: 0.1 µm (*xyz*). **e** 3D plot of cumulative histograms of Bassoon cluster length of all data sets. **f** Corresponding data as box plots. **g** Median values of all data sets plotted against degree of data reduction. Dashed lines indicate the corresponding relative localization density in en bloc scanning in 25 µm sections (see below in **h**) compared to 3D 1 µm sections. Bassoon cluster length remained nearly identical to the original data down to a reduction to 20% of the original localization counts, with no statistical difference between the data sets (Kruskal–Wallis ANOVA *p* = 0.07). **h** Bassoon-cluster localization counts in all imaging conditions. Localization counts decreased from 2D-imaging in 1 µm sections (median ± 25th–75th percentile; 367 ± 258–579) to 3D imaging in 1 µm sections (83 ± 45–187), scanning in 25 µm sections with a scan range of 6 µm (35 ± 15–89), 10 µm (22 ± 13–48) and sequential imaging (22 ± 12–49) highlighting stable length in repeated experiments. **i** Although localization counts per cluster decreased with scanning (and automated evaluation of Bassoon clusters) all imaging conditions gave comparable values of cluster length: 2D (0.41 ± 0.33–0.53 µm), 3D (0.46 ± 0.32–0.66 µm), 6 µm-scans (0.49 ± 0.34–0.721) and 10 µm scans (0.29 ± 0.22–0.43); 10 µm sequential (0.43 ± 0.32–0.63).

**Fig. 7:**
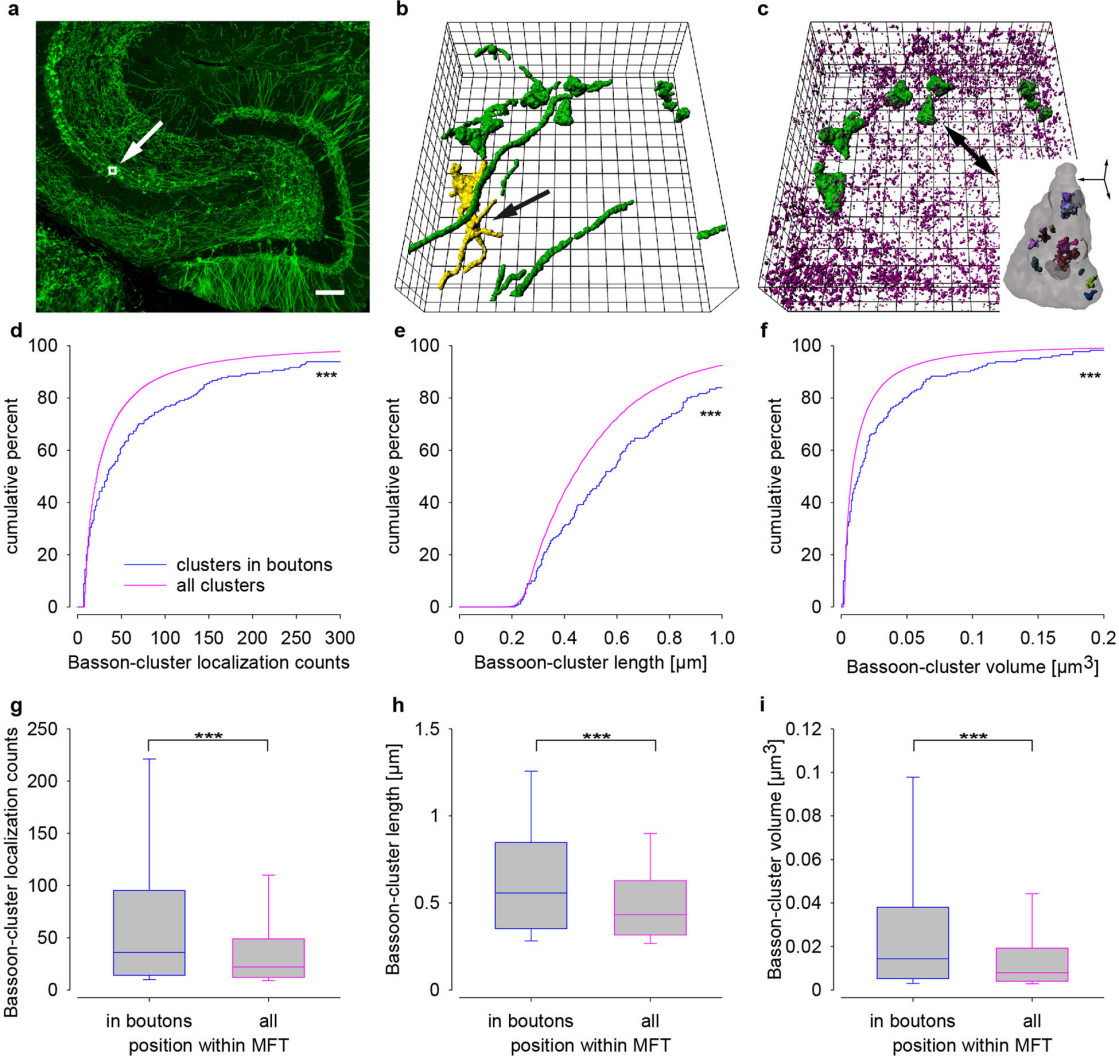
Sequential en bloc 3D imaging in 25 µm thick tissue slices of mEGFP(Ls1) mice showed largest Bassoon clusters in identified mossy fiber boutons. **a** Confocal overview of mossy fibers in a Thy-1 mEGFP(Ls1) mouse with imaging window (25 × 25 µm) depicted to scale. **b** 3D volume reconstruction of mEGFP in *xyz*-view, showing axons and mossy fiber boutons (green), a typical bouton with filopodial extensions is marked in yellow (black arrow). **c** 3D reconstruction of Bassoon (magenta) and mEGFP (green) in large mossy fiber boutons used for calculations of “clusters in boutons” in **d**–**i**; inset depicts the marked bouton (black arrow) with Bassoon clusters (colored). Cumulative plots of localization counts (**d**), cluster length (**e**), and volumes (**f**) comparing all clusters per image (magenta) and those in identified boutons (blue). Corresponding data as box plots **g**–**i**. Scale bar: 100 µm (**a**), grid size 2 µm (**b**, **c**) and scale bar for inset in **c** 1 µm. Asterisks (***p < 0.001) denote statistical significance between the groups.

Data variability led us to question whether the imaging signal to noise ratio was sufficient. To test that labeling efficiency was sufficient we analyzed localization counts per cluster in all imaging conditions used in this paper (Fig. 6). We first compared Bassoon cluster length in high and low-density data from 3D recordings in 1 µm sections (Fig. 6a–f). Low-density data sets were created by reducing localization counts to 90, 80, 66, 50, 33, 20, and 10% of the original data (Fig. 6a–d). Bassoon cluster length remained almost identical down to data sets with only 20% of the original localization counts (Kruskal–Wallis ANOVA *p* = 0.07) (Fig. 6d–g). Stability of Bassoon cluster length was also apparent in our *d*STORM recordings resulting from different imaging conditions. Bassoon cluster length was compared and found to be similar in 2D recordings from thin slices and in 3D scans of thick tissue blocks, even if localization counts per cluster in the latter were lower (Fig. 6h–i). As expected, localization counts per cluster were largest in 2D *d*STORM with a high NA oil-immersion objective (Fig. 6h). Localization density was lower in 3D *d*STORM recordings with a 1.15 NA water-immersion objective in 1 µm sections. The latter on the other hand is clearly necessary to avoid spherical aberrations with thicker samples (Supplementary Fig. S4). For 3D *d*STORM an astigmatic lens was introduced to determine axial position. Criteria for fitting the localization data are stricter resulting accordingly in a lower number of localizations. Nevertheless, Bassoon clusters were clearly resolved as demonstrated by a zoom-in image of the clusters in an en bloc scan (Supplementary Fig. S6). In contrast to the manual evaluation of 2D images, evaluation of 3D images was performed using automated segmentation algorithms that also included smaller clusters consequently further lowering the number of average counts per cluster. Nevertheless, we obtained similar localization counts for Bassoon as reported previously using the same monoclonal anti-Bassoon antibody and a high NA oil-immersion objective^10^. Bassoon cluster length (a parameter that can be obtained from 2D and 3D images) was distributed similarly and the absolute length in all three imaging conditions was similar (Fig. 6i). In 3D *d*STORM scanning the median presynaptic Bassoon cluster density ranged from 2599 counts/µm^3^ in MFBs to 6678 counts/µm^3^ in PP (Supplementary Fig. S5) and the cluster volume from 0.0144 µm^3^ in MFBs (Fig. 7i) to 0.0024 µm^3^ in SC (Fig. 4j). These measurements are compatible with the minimum average density of 8000 localizations/µm^3^, and volumes from 0.002 to 0.03 µm^3^ for paired presynaptic and postsynaptic protein clusters in 3D STORM imaging of dissociated rat hippocampal neurons^12^). Therefore, we conclude that despite lower localization counts in 3D scanning *d*STORM, the method is suited for obtaining precise data from large tissue volumes.

### Sequential scanning 3D *d*STORM of entire, large hippocampal MFBs

The data presented so far cannot be explicitly ascribed to MFBs, since a typical field of view contains contacts of different cell types. Since values for individual boutons are desirable we turned to Thy1-mEGFP(Ls1) mice expressing membrane bound EGFP^38^ for visualization of bouton surfaces. In this preparation, large MFBs were clearly visible as illustrated in a confocal overview of the hippocampal dentate gyrus (Fig. 7a). In a 3D-*d*STORM reconstruction of the anti-GFP fluorescence (Fig. 7b and Supplementary Video 2) MF boutons, filopodia and axons are illustrated. For further experiments brain slices were mounted on fluorescent bead-coated coverslips, which allowed to align sequential images and to correct for drift (Supplementary Figs. S2 and S3). We first visualized Bassoon with the monoclonal mouse anti-Bassoon and Alexa647-conjugated anti-mouse secondary antibodies. After washing we stained the tissue with Alexa647-conjugated anti-GFP nanobodies (Supplementary Video 2). Repeated fast axial scanning (ten times) in the middle of the 25 µm brain slice through the 10 µm region of interest showed homogeneous distributions of localization intensity and counts (Fig. 7c and Supplementary Fig. S3). Drift was reduced by starting measurements via remote control from outside the laboratory after sample stabilization, and by imaging beads at the beginning and end of each scan while total acquisition time was about 3 h per experiment. Localization counts, cluster length and volume of Bassoon clusters could be assigned to MFBs (median ± 25th–75th percentile: 36 ± 14–96 counts; length 0.558 ± 0.352–0.848 µm, volume 0.0144 ± 0.0052–0.0380 µm^3^, *n* = 181) and were larger (*p* < 0.001) than those of Bassoon clusters in the whole imaging window (counts: 22 ± 12–49; length 0.433 ± 0.315–0.629 µm; volume 0.0080 ± 0.0040–0.0193 µm^3^, *n* = 35,632) (Fig. 7d–i). Bassoon clusters in MFBs (2599 ± 2388–2896 counts/µm^3^) were less dense (*p* < 0.001) than those in the whole imaging window (2744 ± 2499–3055 counts/µm^3^) (Supplementary Fig. S5d, g). Twenty-one individually identified MFBs showed different volumes and number of clusters per bouton with on average nine clusters per large MFB (Supplementary Table 1). The mean MFB-volume was 7.9 µm^3^ (ranging from 1.3 to 32 µm^3^). The biggest MFB also contained the largest number of Bassoon clusters and cluster number increased with MFB volume (Spearman’s *r* = 0.74212, *p*(two-tailed) = 0.00012). Bassoon cluster size did, however, not increase with MFB volume. In summary sequential *d*STORM-imaging enabled volumetric protein cluster mapping in identified large MFBs.

## Discussion

We used targeted volumetric *d*STORM to compare synaptic contacts in defined regions of mouse hippocampus at nanoscopic resolution. 2D-imaging thin sections revealed highly significant larger Bassoon cluster length along the ventro-dorsal axis of the hippocampus in eight adult male mice. The two levels in Fig. 2 correspond to the distance between the upper and the lower surface of adjacent acute hippocampal slices in electrophysiological recordings^25,26^, and the observed differences in cluster size likely contribute to the typically observed functional variability.

We detected the substantial variability in Bassoon cluster size within individual images from one section. To minimize confounding factors, such as overlapping projections in 2D-imaging and mechanical or optical truncation we turned to 3D-*d*STORM and imaged a 30 × 30 µm field of view with typically more than 20 synaptic contacts in a single focal plane. Astigmatic imaging with the red fluorophore Alexa Fluor 647 and a water immersion objective allowed imaging in 25 µm thick sections. To control that labeling efficiency was sufficient we analyzed localization counts per cluster in all imaging conditions (Fig. 6). As expected, largest counts per cluster were found in 2D *d*STORM with a high NA oil-immersion objective. Counts were lower in 1 µm sections in 3D *d*STORM using a 1.15 NA water immersion objective because of the stricter fitting criteria for 3D localization. Furthermore, in contrast to manual evaluation of the 2D data, evaluation in 3D was performed using automated segmentation algorithms. These algorithms included also smaller clusters and thus further reduced counts per cluster. Nevertheless, similar number of localizations for Bassoon were obtained here as published in Fig. S4 in Dani et al.^10^. Counts and intensity/local background were not influenced by imaging depth but remained constant (Supplementary Fig. S3). Counts depend on conditions such as labeling efficiency, degree of labeling of the secondary antibody, buffer composition, illumination, NA of the objective, camera gain and number of frames recorded^14,39^. Taking our 2D-data as a reference, we found that Bassoon cluster length (a parameter that can be obtained in 2D and 3D imaging) shows similar distributions and absolute size in all imaging conditions (Fig. 6). Furthermore, the density and volume of Bassoon clusters in our 3D scanning data are comparable with those defined for synaptic clusters in cultured hippocampal neurons^12^. Thus, despite lower counts in 3D *d*STORM, this method is well suited for obtaining precise data from large tissue volume.

En bloc 3D *d*STORM has, from a biological perspective, the advantage that it delivers images of non-truncated structures. This is important, e.g., for accurate size determination of AZs in the MFT (Figs. 3–5). Furthermore, it is a prerequisite for obtaining reliable numbers of AZs in entire structures such as large MFBs. We measured Bassoon cluster number per MFB (mean 8.6 range 1–45). Mean bouton volume (7.9 µm^3^) and the range of volumes (1.3–32 µm^3^) given in Supplemental Table 1 fit reasonably to a mean volume of 13.51 µm^3^ with range 10–19 µm^3^ given in Wilke et al.^6^ (Table 1) using block face scanning and the 4.9 and 17.4 µm^3^ reported for two reconstructed MFBs in Zhao et al.^24^ using serial thin sectioning EM reconstruction.

We determined highly significant different protein clusters in neighboring synaptic circuits within a single anatomically preserved tissue block of mouse hippocampus avoiding cutting and tissue processing artifacts (Fig. 4). Median Bassoon clusters in MFT were larger but less dense compared to Schaffer collaterals or perforant path (Fig. 4i, j and Supplementary Fig. S5e). Due to the large number of samples and the inherent comparability of this type of measurement within a tissue block; this approach should also be useful for measurements in other tissues and other brain regions.

Despite standardized procedures no clear evidence for Forskolin induced plasticity was obtained (Fig. 5). The effect of Forskolin treatment was different in each animal with similar control values for cluster size and large samples per animal. Since Forskolin induces dramatic functional effects in MFBs^37^ this result was unexpected. However, a recent study using hippocampal slice cultures, STED microscopy, high pressure freezing, and tomography also reported no significant changes in AZ number and length in MFBs after Forskolin stimulation^18^. In contrast an earlier study using a more drastic stimulation reported significant increases in AZ number and reductions in AZ size^24^. AZ length for controls in Fig. 4g in Maus et al.^18^ of about 370 nm and of 222 nm in Fig. 6 in Zhao et al.^24^ are somewhat lower than the length of Bassoon clusters for controls in our Fig. 5 (on average 488 nm for the three animals). Bassoon cluster length is quite obviously a different parameter than electron density of AZs in EM. However, Bassoon is a large protein^31^ that may well extend beyond the electron dense area. The linkage error with primary and secondary IgG antibodies in standard immunolabeling of about 17.5 nm also needs to be considered^40^, which likely contributes to the somewhat larger values in our study. Furthermore, truncation needs to be considered in thin sections used for EM. Therefore, correlative light and electron microscopy of AZs with immunostaining for Bassoon will ultimately be necessary to further quantify the relationship of these AZ parameters.

With sequential staining it was possible to image two proteins with Alexa Fluor 647^36,41–43^, the most reliable *d*STORM dye for quantitative imaging^20,39^. Using sequential staining and en bloc 3D imaging we were able to characterize the geometry and size of protein clusters and also map clusters to identified MFBs. Since Bassoon was always imaged first and then GFP the second round of imaging did not interfere with the quantification of Bassoon cluster sizes. The clusters in MFBs were significantly larger than the clusters recorded in the whole region of interest and with mice expressing membrane bound EGFP, it was possible to distinguish between contacts formed by the MFB itself and its GABAergic filopodial extensions^29,38,44^. Presynaptic structures, e.g., from interneurons likely contributed to the smaller clusters in our images for Figs. 4 and 5^45^.

The large size and highly complex geometry of MFBs with on average less than 0.5 µm from one synaptic contact to the next in EM^5,6,24^ represents a major imaging challenge further complicated by the highly variable size of individual contacts. The size of the individual clusters in 3D images of Bassoon falls within the range expected for AZs based on previous EM work^5,7,18,24^. The approach described here can be used to map the 3D distribution of any protein in thick tissue slices for which highly specific antibodies are available. While we used mice expressing membrane bound EGFP to identify MFBs the described method can likewise be modified to correlate function, and molecular organization of individual synapses in acute slices or even in vivo by filling neurons with fluorescent dyes for identification and subsequent labeling and volumetric 3D-*d*STORM imaging.

## Methods

### Animals

Male C57/BL6, Thy1-EGFP(M), and Thy1-mEGFP(Ls1)^38^ mice on C57/BL6 background were used (Fig. 2: eight 24-week old Thy1-EGFP(M) and one 12-week old C57/BL6, Figs. 3 and 4: one 12-week old Thy1-EGFP(M), Figs. 6 and 7: each three 12-week old mice). Animal procedures were approved and performed in accordance with the guidelines of institutional and regulatory authorities (Permit number RUF-55.2.2-2532-2-572-16 of the district government of Lower Franconia), the EU Directive 2010/63/EU and the United States Public Health Service’s Policy on Humane Care and Use of Laboratory Animals.

### Cryosectioning

The mice were sacrificed with CO_2_ and perfused transcardially with phosphate buffered saline (PBS) followed with 4% paraformaldehyde (PFA) in 0.2 M PBS. Brains were removed, post-fixed in 4% PFA overnight at 4 °C, dehydrated in 30 % sucrose solution and rapidly frozen. One micrometer of thin horizontal cryosections were obtained using a cryotome (Leica CM 3050, Leica, Wetzlar, Germany) in a standardized manner. To obtain a uniform cutting angle for horizontal brain sections, brains were mounted with the dorsal cortical surface facing down on aluminum carriers. Using the lower edges of both cortices and the pons as landmarks to align the specimen to the blade, the brains were trimmed until lateral ventricles were opened and the characteristic double C-shape of the hippocampus could first be identified at both sides. From this base level 100 µm of the brain were cut in 1 µm thin slices on silan-coated coverslips and the following 100 µm were cut in 10 µm thick sections on regular microscope slides. To align the cutting level between different animals on the identical hippocampal structure, we used images from Nissl stained horizontal brain atlas (brainmaps.org)^46^ and aligned the sections according to the shape of the dentate gyrus. Cryosections between 900 and 1000 µm corresponding to the image 141 of the brain atlas are referred to as level 1 and sections between 1500 and 1600 µm corresponding to image 117 of the atlas as level 2. For en bloc 3D *d*STORM imaging, horizontal 25 µm thick cryosections were cut into 12-well tissue culture plates for staining free-floating and mounted after the last washing step on fluorescent beads coated coverslips (see below).

### Silan-coated coverslips

Round shaped, 18 mm diameter, high precision coverslips (Marienfeld No 1.5H, Lauda-Königshofen, Germany) were mounted vertically in a custom-made comb-like PTFE-holder, immersed with 3-aminopropyl-triethoxysilane (Sigma 440140, Sigma-Aldrich, Schnelldorf, Germany) 2% v/v in methanol for 2 min, washed shortly with methanol 100% and distilled water and dried. For sequential *d*STORM imaging coverslips were coated with FluoSpheres®, 0.2 µm, orange fluorescent (540/560) (Thermo Fisher Scientific, Darmstadt, Germany, dilution 1:1000). FluoSpheres for determination of *x*, *y*, and *z* position and correction of lateral and focal drift were diluted in PBT 0.125%. Seventeen microliter were spread on silan-coated coverslip over an area of approximately 1.6 cm^2^, dried, washed with destilled water, and dried again.

### Preparation of acute hippocampal slices and induction of chemical plasticity

Twelve acute hippocampal slices from three 8 week-old, male Thy1 mEGFP(Lsi1) mice were prepared as described in Hallermann et al.^25^. The mice were sacrificed under deep isofluorane anesthesia by decapitation in accordance with institutional guidelines. The head was dropped into ice-cold cutting solution (artificial cerebrospinal fluid = aCSF, composition described below, with 75 mM sucrose). The brain was then quickly removed into ice cold carbogenated cutting solution and cut into two transverse 300-µm-thick slices at definite hippocampal levels (1500 and 1800 µm from the ventral brain surface) using a vibratome (Leica VT1200) with the right and left hemispheres separated. For incubation of the slices in custom-made incubation chambers, aCSF containing 125 NaCl, 25 NaHCO_3_, 2.5 KCl, 1.25 NaH_2_PO_4_, 25 glucose, 2 CaCl_2_, and 1 MgCl_2_ (in mM), equilibrated with 95% O_2_/5% CO_2_, (pH 7.3) was used. Slices were first incubated at 35 °C for 30 min and subsequently held at room temperature. For induction of plasticity, the slices were exposed to 50 µM Forskolin (F6886, Sigma) in 0.5% DMSO/aCSF or to 0.5% DMSO in carbogenated aCSF for 30 min at room temperature. Before fixation the slices were transferred into separate maintenance chambers containing carbogenated aCSF and kept at room temperature for 30 min before fixation.

#### Fixation, flat-mounting, and cryosectioning of acute slices

The 300 µm thick brain slices were fixed with 1 ml of cold 4% paraformaldehyde in phosphate buffer for 2 h and washed three times (each time for 5 min) with phosphate-buffered saline (PBS). The slices were placed into sucrose (30%) PBS overnight, flat mounted on specimen holders with cryogel (Cryo-Gel 39475237, Leica) using a custom-made mounting devise and kept in −80 °C for 12 h. Using a cryotome each 300 µm thick hippocampal slice was then cut into three 25 µm thick sections, which were placed into 12-well cell culture plates (Corning) containing PBS for staining free-floating.

### Immunofluorescence

#### Antibodies used

Primary antibodies were used in the following concentrations: mouse monoclonal antibody (mAb) anti-α-Bassoon (Enzo Sap7F407, Enzo Life Sciences, Lörrach, Germany, 1:500), rabbit polyclonal antibody anti-α-Homer 1 (Synaptic Systems, Göttingen, Germany, 106002, 1:500), rabbit polyclonal and mAb anti-Zinc transporter 3 (ZnT3) (Synaptic Systems 197002 and 197011, 1:500), anti GFP nanobodies (GFP-Trap® (uncoupled protein), ChromoTek, Munich, Germany, custom conjugation with Alexa647^47^, 1:1000).

Secondary antibodies were used in the following concentrations: Alexa 647 conjugated goat α-mouse antigen binding fragment (Fab2), Alexa 532 conjugated goat α-mouse Fab, Alexa 647 conjugated goat α-rabbit Fab, Alexa 532 conjugated goat α-rabbit Fab (Invitrogen, Thermo Fisher, Darmstadt, Germany,1:500 each).

PEPperMAP® Type 1 Epitope Mapping of the mouse monoclonal anti-Bassoon antibody against two undisclosed antigens was performed with 15 amino acid antigen-derived peptides with a peptide-peptide overlap of 14 amino acids by PEPperPRINT GmbH (Heidelberg, Germany). The antigen-derived peptide microarrays were incubated with the antibody samples at concentrations of 1 µg/ml and 10 µg/ml in incubation buffer followed by staining with the secondary antibodies goat anti-mouse IgG (H+L) DyLight680 or sheep anti-rabbit IgG (H+L) DyLight680 and read-out with a LI-COR Odyssey Imaging System. Quantification of spot intensities and peptide annotation were done with PepSlide® Analyzer (PEPperPRINT).

Controls for antibody staining quality were performed as described below using 1 µm hippocampal brain sections (Supplementary Fig. S7).

#### Staining protocol for 1 µm sections on coverslips

The sections were washed with 0.02 M glycine (Sigma) in phosphate buffered saline (PBS) for 30 min to quench free aldehyde autofluorescence, blocked with blocking solution consisting of 1% bovine serum albumin (BSA) (Sigma) and 5% normal goat serum (NGS) (Seralab, West Sussex, UK) in 0.3% PBT (PBS containing 0.3% Triton X-100, Sigma) for 90 min and incubated with primary antibodies at 4 °C overnight. Samples were washed with blocking solution twice for 5 and twice for 20 min, followed by incubation with secondary antibodies for 2 h at room temperature. After identical washing steps, sections were kept in 1× PBS at 4 °C until imaging.

#### Staining protocol for free floating 25 µm sections

The sections were washed with 0.02 M glycine (Sigma) in PBS for 2 h, blocked with blocking solution consisting of 1% BSA (Sigma) and 5% NGS (Seralab) in 0.3% PBT (PBS containing 0.3% Triton X-100, Sigma) overnight and incubated with anti-α-Bassoon (Enzo Sap7F407, 1:500) antibodies at 4 °C for 36 h. Sections were then washed with blocking solution twice for 5 min and twice for 20 min, followed by incubation with Alexa 647 conjugated goat anti-mouse Fab fragment (Invitrogen, 1:500) at 4 °C for 24 h. Identical washing steps were then repeated and the sections kept in PBS until they were mounted onto silanized, beads-covered coverslips, quickly dried, and kept in 1× PBS at 4 °C until 3D *d*STORM imaging.

#### Staining protocol for sequential imaging

For sequential staining with the strategy adapted from multitarget fluorescence imaging^36,41–43^ free floating 25 µm hippocampal sections were washed for 30 min in 0.02 M glycine (Sigma) and 0.3% Triton X-100 (Sigma) in 1× PBS to quench free aldehyde autofluorescence, blocked overnight with blocking solution consisting of 1 % bovine serum albumin (BSA) (Sigma) and 5% normal goat serum (NGS) (Seralab, West Sussex, UK) in 0.3% PBT (1xPBS containing 0.3% Triton X-100, Sigma): The sections were then incubated at 4 °C for 36 h with mouse monoclonal anti α-Bassoon (Enzo, Sap7F407) antibodies at a dilution of 1:500 in blocking solution, washed with blocking solution twice for 5 and twice for 20 min, followed by incubation with the Alexa 647 goat anti-mouse F(ab’)2-fragment (Invitrogen) for 24 h at 4 °C and washed as described above. After the last washing step, the sections were mounted on beads-covered coverslips, quickly dried, and kept in 1× PBS at 4 °C until imaging. First imaging was performed as described later. For the sequential imaging sections on cover slips were washed after first imaging step with 1× PBS and kept in 1× PBS at least for 6 h prior to incubation with anti-GFP nanobodies at 4 °C for 24 h, washed two times for 5 min and twice for 20 min with blocking solution and kept in 1× PBS at 4 °C until second imaging.

### Imaging

#### *d*STORM (direct stochastic optical reconstruction microscopy)

Cover slips with stained brain slices were mounted in a custom-made holder and incubated in imaging buffer (100 mM mercaptoethylamine (MEA, Sigma) in PBS, buffered at pH 7.8–7.9) to allow reversible switching of single fluorophores during data acquisition. Images were acquired using an inverted microscope (Olympus IX-71, Olympus, Hamburg, Germany, with Olympus APON60XOTIRF 60×, NA 1.45, oil immersion or Zeiss LD C-Apochromat, Zeiss, Jena, Germany, 63×, NA 1.15 water immersion objective) equipped with a nosepiece-stage (IX2-NPS, Olympus). 644 nm (iBEAM-SMART-640-S, Toptica, Gräfelfing, Germany) and 532 nm (Qioptiq Nano 250-532, Qioptiq; Asslar, Germany) lasers were used for excitation of Alexa Fluor 647 and Alexa Fluor 532, respectively. Laser beams were passed through a clean-up filter (Brightline HC 642/10, Semrock, and ZET 532/10, Chroma, AHF Analysetechnik, Tübingen, Germany, respectively) and two dichroic mirrors (Laser-MUX BS 514–543 and HC-quadband BP, Semrock) onto the sample. The emitted fluorescence was filtered with a quadband-filter (HC-quadband 446/523/600/677, Semrock) and divided onto two cameras (iXon Ultra DU-897-U, Andor, Acal BFi, Grübenzell, Germany) using a dichroic mirror (HC-BS 640 imaging, Semrock). In addition, fluorescence was filtered using a longpass-filter (Edge Basic 635, Semrock) or bandpass-filter (Brightline HC 582/75, Semrock) for red and green channels, respectively. Pixel sizes were 126 nm (red) and 128 nm (green). Single fluorophores were localized and high resolution-images were reconstructed with rapi*d*STORM (10 nm /pixel sub-pixel binning)^48^ (www.super-resolution.de).

#### 3D *d*STORM

3D *d*STORM images were obtained at a setup mainly as described above with a Zeiss 63×, NA 1.15 water immersion objective, a Piezo z-stage (P-736.ZR 2, Physik Instrumente, Karlsruhe, Germany) and a cylindrical lens in the emission path to obtain information about the three dimensional position of the fluorophore^49^. For z-calibration of the microscope, we used multi-fluorescent beads (100 nm TetraSpeck, ThermoFisher) adsorbed on a coverslip and covered with water. The calibration sample was axially moved at constant speed through the focal plane by the piezo and the widths of the PSF in *x* and *y* is evaluated with rapi*d*STORM 3.3.1^48^ (www.super-resolution.de). The interpolations of the widths against the known z position is performed using cubic B-splines and serve as calibration table^50^. Axial position of localizations in samples was determined using rapidSTORM as previously described^50^.

#### Sequential en bloc 3D *d*STORM

A graphical overview of sequential en bloc scanning workflow is depicted in Supplementary Fig. S2. For scanning sequential 3D-*d*STORM a 647 nm Laser (F-04306-113, MPB Communications Inc., Pointe-Claire, Quebec, Canada) was used. For imaging Alexa647 with long term pH stability at pH 7.8 we used a reducing agent 2-mercaptoethylamine-hydrochloride (MEA, 100 mM, Sigma) in a 0.2 M sodium phosphate buffer and an oxygen-scavenging system (10% (wt/vol) glucose, 10 U/ml glucose oxidase and 200 U/ml catalase. Samples on coverslips with fluorescent beads were placed in a custom build imaging chamber and mounted on the microscope with 14 µl of immersion media (Immersol W (2010), Zeiss, Jena, Germany). To minimize drift measurements were started via remote control from outside the laboratory 45 min after the region of interest was defined. Each measurement consisted of several videos with 15,000 frames at 100 Hz each. In order to reconstruct larger volumes, we used the piezo for continuous axial scanning over the 10 µm *z*-range of the ROI during one movie (except for imaging Forskolin treated sections, where a *z*-range of 6 µm was used). To counter effects of out of focus photobleaching we performed repeated scanning: typically, ten scans were performed inverting the direction of the movement after each scan. The measurement sequence was controlled by a custom-built micromanager^51^ plugin. For sequential imaging samples were washed with PBS and the second staining procedure was performed as described above. Before and after each measurement fluorescent beads at the surface of the coverslip were imaged for alignment of sequential scans and drift correction. Precise position of beads was determined with rapi*d*STORM 3.3.1 and alignment of sequential stacks was performed using elastic transformations obtained with ImageJ^52^ plugin bunwarpJ^53^.

### Data evaluation

Raw localization data obtained from rapi*d*STORM 3.3.1 were examined and further processed with FIJI^54^ (Figs. 2, 3, and 5) and Imaris (Bitplane, Zürich, Switzerland) (Figs. 4, 5, and 7, Supplementary Figs. S5 and S6).

#### Data evaluation for 2D imaging

To reflect the complex shape of Bassoon clusters, area measurements were performed using the freehand ROI tool, length and width measurement were performed using the freehand line respectively line tool in FIJI. To obtain an estimate for relative protein content within an AZ we measured localization counts per AZ, i.e., the total number of stochastic fluorophore blinking events per cluster. Using standardized imaging conditions the number of localization counts depends on the number of fluorophores present at the target structure, which in turn depends on the number of bound secondary and primary antibody and thereby on the number of epitopes^20^.

#### Data processing and analysis for sequential en bloc 3D *d*STORM

To avoid focal drift we allowed the sample to settle on the microscope for 45 min prior to recording. Fluorospheres attached to the coverslip were imaged before and after each scanning recording and the precise position of the beads in *x*, *y*, and *z* was determined with rapi*d*STORM 3.3.1. Using custom written Python code (language versions 2.7 and 3.5) precise localization data of beads before and after the scanning recording was used to estimate and correct lateral and focal drift.

We used custom Python scripts to prepare the rapi*d*STORM localization files for further processing with Imaris (Bitplane). Localization tables from individual scans were concatenated and true *z* position of each localization was calculated using information about the *z*-position of the piezo during scanning. Drift correction was applied using rigid transformations obtained from bead recordings before and after each measurement. Finally, images were filtered using density-based clustering (DBSCAN) in sckit-learn^55^ in Python to remove noise. After individually processing both channels in sequential scans the two channels were combined and loaded into Imaris via the “Super Resolution Localization Data To Image – XTension” and the “Super Resolution Localization Data To Spots – XTension”. Once loaded, isosurfaces created by the Imaris surface module for volume data extraction were used to detect Bassoon clusters and to reconstruct mossy fiber boutons. For further data analysis a lower cut-off of eight localizations was used for filtered data (see histogram in Supplementary Fig. S8). Boutons were separated manually from supporting axons. Identification of Bassoon clusters belonging to one bouton was done using Imaris XTension distance transformation. All Bassoon clusters with a center of mass inside a reconstructed super-resolved bouton signal were considered to belong to this bouton.

#### Aberration and background

To avoid spherical aberration imaging in thick samples was performed as described above. Using water immersion objectives and TetraSpeck Microspheres (100 nm, T-7279, Life-Technologies beads 1:200 in Matrigel matrix, phenol-red free, Corning) we demonstrate aberration free recording up to a depth of 20 µm in the sample (Supplementary Fig. S4). In our hands brain sections behave like aqueous samples, as the sections have undergone a long permeabilization process with Triton X-100 over several days (see above the methods for immunofluorescence) and are submersed in an aqueous imaging buffer (see “Methods” section for immunofluorescence, *d*STORM). Spherical aberration would lead to distorted PSFs and thus to a gradient in localization intensity and localization density. Using en bloc 3D scanning we can show a homogenous localization intensity and localization density throughout the imaging volume (Supplementary Fig. S3a, b) without severe spherical aberration.

Using epifluorescence illumination in thick samples light scattering and out of focus light will contribute to fluorescence background. Using a localization intensity threshold of 10000 arbitrary units in raw data analysis with rapi*d*STORM, we achieve a high signal to noise ratio that is homogenously distributed throughout the imaging window (Supplementary Fig. S3c, localization intensity/local background of 11 ± 8–17, median ± 25th and 75th percentile).

#### Creation of datasets with artificially reduced localizations counts

Raw data from 3D recordings in 1 µm sections was filtered for background localizations as described above. Data sets with reduced localization density were created from background subtracted localization tables by omitting every 10th (90%), 5th (80%), 3rd (66%), or 2nd (50%) localization, and by using every 3rd (33%), 5th (20%), or 10th (10%) localization, respectively. All data sets were combined and loaded into Imaris as separate channels. Segmentation of all channels was performed separately, and the isosurface threshold was adjusted proportionally to the amount of data reduction, i.e., the threshold value for the dataset with 80% of original localizations was reduced to 80% of the threshold used for the original data. Segmentation of data with only 10% of original data showed a fraction of clusters with a typical length of 0.158 µm originating from a single localization. A minimal threshold of 0.160 µm in length was used for further analysis of Bassoon cluster length.

### Statistics and reproducibility

Statistical analyses were performed with Sigma Plot 12 and 14 (Systat Software GmbH, Ekrath, Germany) using the non-parametric Mann–Whitney rank sum test or the non-parametric ANOVA for multiple comparisons. Asterisks indicate the significance level (*p < 0.05, **p < 0.01, ***p < 0.001). Data are reported as median ± 25th and 75th percentile for non-parametric data unless indicated otherwise and as mean ± SD for parametric data.

### Reporting summary

Further information on research design is available in the Nature Research Reporting Summary linked to this article.

## Supplementary information

Supplementary Information

Description of Additional Supplementary Files

Supplementary Video 1

Supplementary Video 2

Supplementary Data 1

Supplementary Data 2

Supplementary Data 3

Supplementary Data 4

Supplementary Data 5

Supplementary Data 6

Supplementary Data 7

Supplementary Data 8

Supplementary Data 9

Supplementary Data 10

Reporting Summary

## Data Availability

The data sets generated during and/or analyzed during the current study are available from the corresponding authors on reasonable request. The source data underlying Fig. 2g–j are provided as Supplementary Data 1, the source data underlying Fig. 3g–h as Supplementary Data 2, the source data underlying Fig. 4e–j as Supplementary Data 3, the source data underlying Fig. 5a-i as Supplementary Data 4, the source data underlying Fig. 6e–i as Supplementary Data 5, the source data underlying Fig. 7d–i as Supplementary Data 6, the source data underlying Supplementary Fig. S1c, e–g, i–k are provided as Supplementary Data 7, the source data underlying Supplementary Fig. S3a–c as Supplementary Data 8, the source data underlying Supplementary Fig. S5a–g as Supplementary Data 9 and the source data underlying Supplementary Fig. S8 as Supplementary Data 10.

## Code availability

Software used for data analysis in this study included Rapid STORM 3.3.1., FIJI-ImageJ, Imaris-Bitplane 9.2., Python 2.7., Python3.5, Python 3.6, Sigmaplot 12, and 14 (Systat). The custom Python codes are available from the corresponding authors on reasonable request.
