# Supplementary material for: Targeted volumetric single-molecule localization microscopy of defined presynaptic structures in brain sections
Source: Commun Biol. 2021 Mar 25;4:407. doi: 10.1038/s42003-021-01939-z (PMC7994795; doi:10.1038/s42003-021-01939-z)
Supplement: Supplementary file 1 — Supplementary Information [file 42003_2021_1939_MOESM1_ESM.pdf]

## Supplementary Figures with legends, Supplementary Table

# Targeted volumetric single-molecule localization microscopy of defined presynaptic structures in brain sections

Martin Pauli [1,2*], Mila M. Paul [1*], Sven Proppert [1,2*], Achmed Mrestani [1*], Marzieh Sharifi[1,2], Felix Repp[1,2,3], Lydia Kürzinger[1,2], Philip Kollmannsberger[3], Markus Sauer (m.sauer@uni-wuerzburg.de)[4,5], Manfred Heckmann (heckmann@uni-wuerzburg.de)[1, 5], Anna-Leena Sirén (Siren_A@ukw.de)[2, 5]

[1]Institute for Physiology, Department for Neurophysiology, Julius-Maximilians-University Würzburg, 97070 Würzburg, Germany

[2]Department of Neurosurgery, University Hospital of Würzburg, 97080 Würzburg, Germany

[3]Center for Computational and Theoretical Biology, Julius-Maximilians-University Würzburg, 97074 Würzburg, Germany

[4]Department of Biotechnology and Biophysics, Biocenter, Julius-Maximilians-University Würzburg, 97074 Würzburg, Germany

[5]Corresponding authors

* These authors contributed equally.

**Supplementary Fig. S1. Peak-to-peak-distance, Bassoon cluster length, width and localization counts in synaptic contacts in side and plane view.**

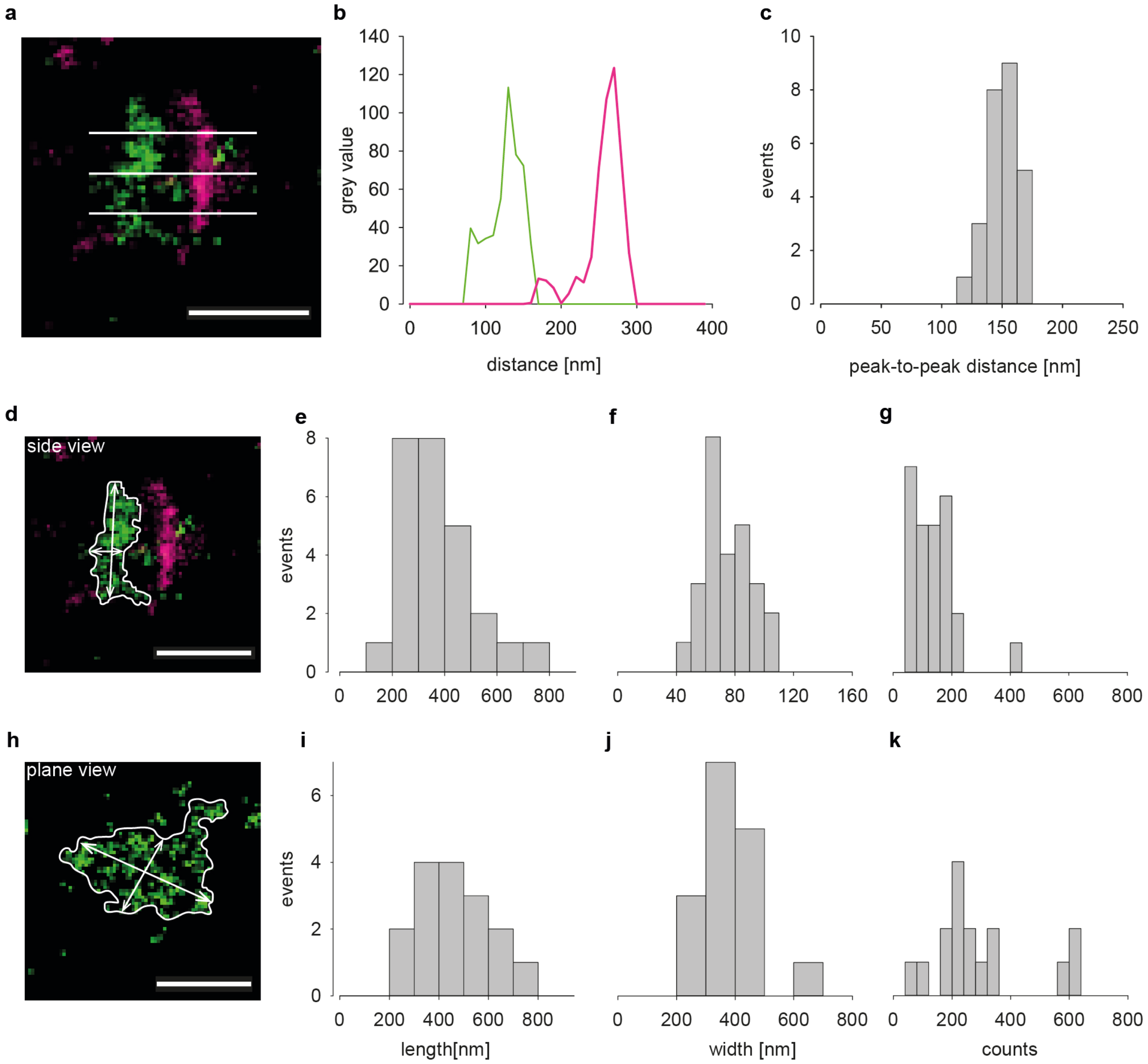


**a** Representative image of a contact in side view with presynaptic Bassoon in green and Homer1 in magenta. White lines depict positions used for peak-to-peak measurements between pre-and postsynaptic protein clusters. **b** Grey values plotted against distance for peak-to-peak measurements between Bassoon (green) and Homer1 (magenta, measurement corresponds to central line in **a**). **c** Histogram of peak-to-peak distances for 26 contacts in one section. **d** Representative *d*STORM image of contact in side view, Bassoon (green), Homer 1 (magenta). White line outlines Bassoon area. Arrows illustrate measurements of length and width. Histograms of length **e**, width **f** and localization counts **g** of 26 Bassoon clusters in side view. **h** Representative *d*STORM image of a contact in plane view, Bassoon (green; Homer 1 not shown for clarity). Histograms of length **i**, width **j** and localization counts **k** of 16 Bassoon clusters in plane view. Scale bars in **a**, **d** and **h** 300 nm.

**Supplementary Fig. S2. Setup for *en bloc* 3D imaging with axial scanning and flow chart for data analysis.**

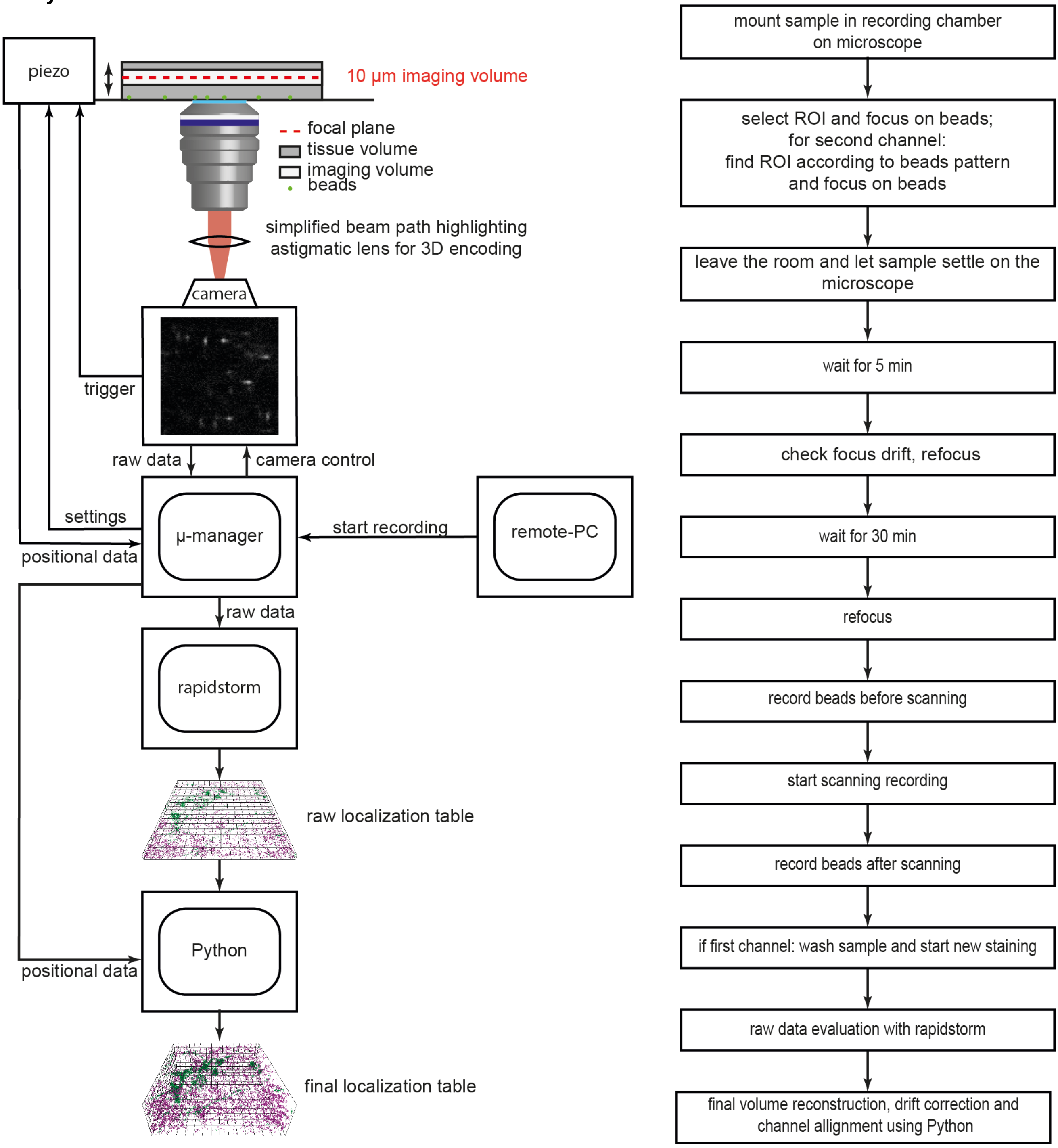


On the left a schematic illustration of the setup with a 25 µm thick brain section on fluorescent beads (green)-coated coverslip (not to scale). During axial scanning, the relative position of the focal plane (red dotted line) is continuously stepped up and down (black arrow) through the region of interest (white). Piezo and cameras are controlled with micromanager and recordings started from a remote PC after stabilization of the setup. Localization tables of raw data are created using rapi*d*STORM and together with positional data from micromanager directly loaded and analyzed with custom written Python code and the web-based Python interface Jupyter. On the right a flow-diagram of the individual steps during image acquisition and data analysis.

**Supplementary Fig. S3. Homogeneous distribution of AD-counts, density of localizations and signal-to-local background ratio during *en bloc* 3D continuous axial scanning in thick tissue slices in measurements of fluorescence with mouse-anti-Bassoon monoclonal antibody and secondary Alexa647-Fab2.**

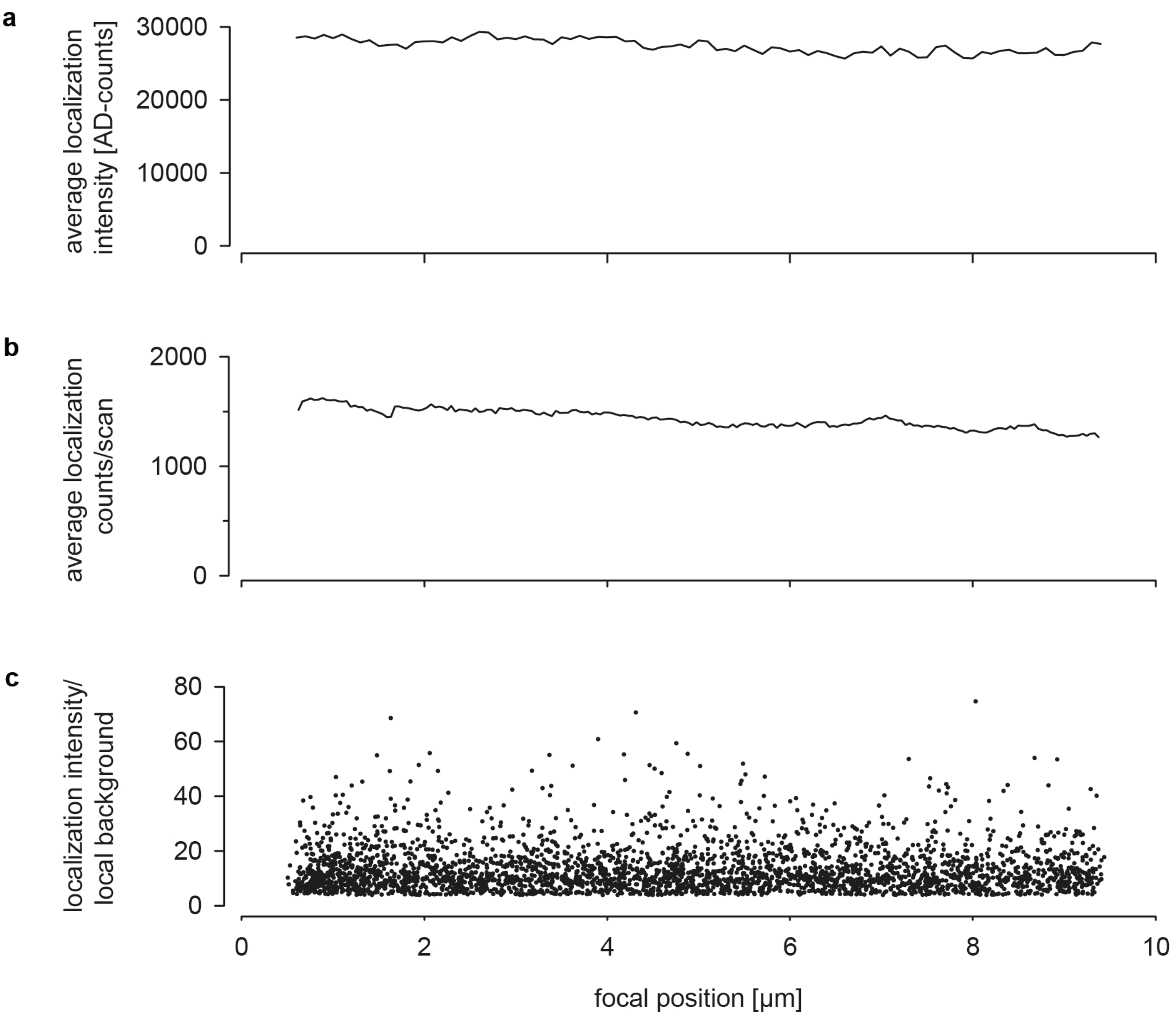


**a** Average localization intensity in one scan, **b** average localization counts per scan of all axial scans in one image and **c** localization intensity/local background relative to focal position for region of interest (see methods). For clarity, only every hundredth data point is plotted in **c**. Average localization intensity/local background for all data in one image was 11 ± 8 -17 (median ± 25th and 75th percentile; n = 309350).

**Supplementary Fig. S4. Spherical aberration at variable imaging depths.**

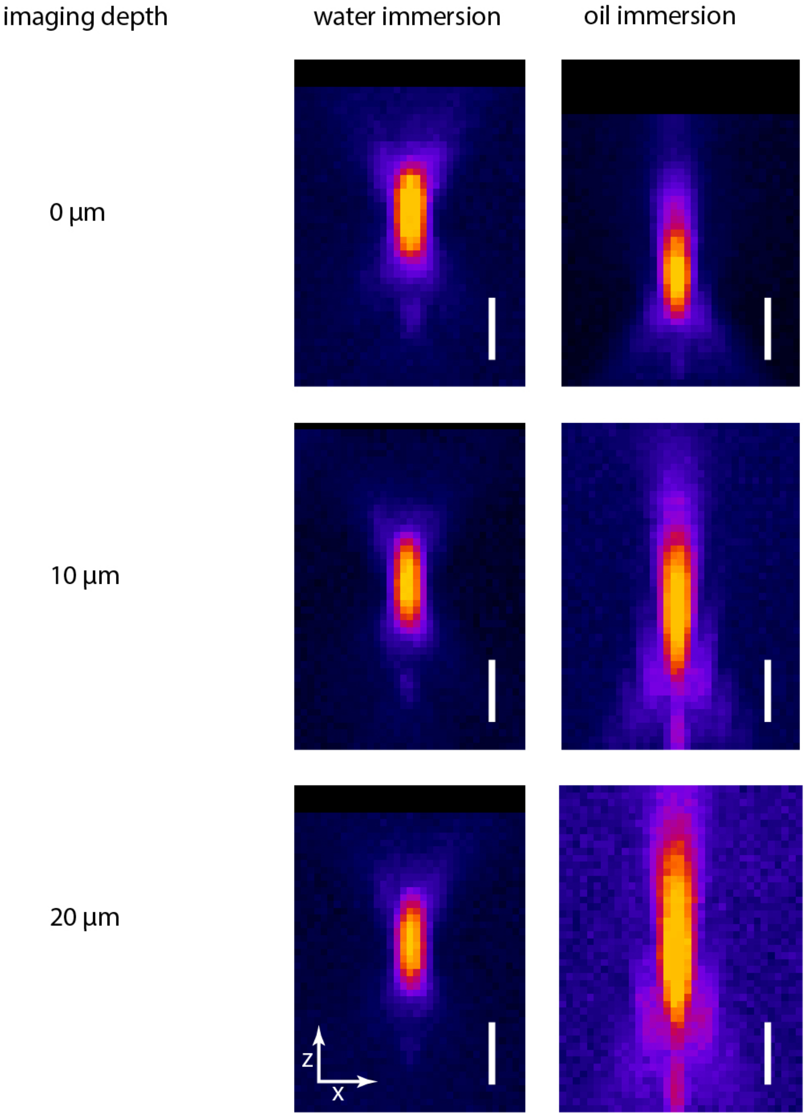


Experimental point spread functions (PSFs) of water and oil-immersion lenses at variable depths obtained by imaging fluorescent 100 nm TetraSpeck beads embedded in hydrogel with refractive index n ≈ 1.34 (Matrigel, BD Bioscience). Scale bar = 1 µm.

**Supplementary Fig. S5. Bassoon-cluster density in 3D scanning.**

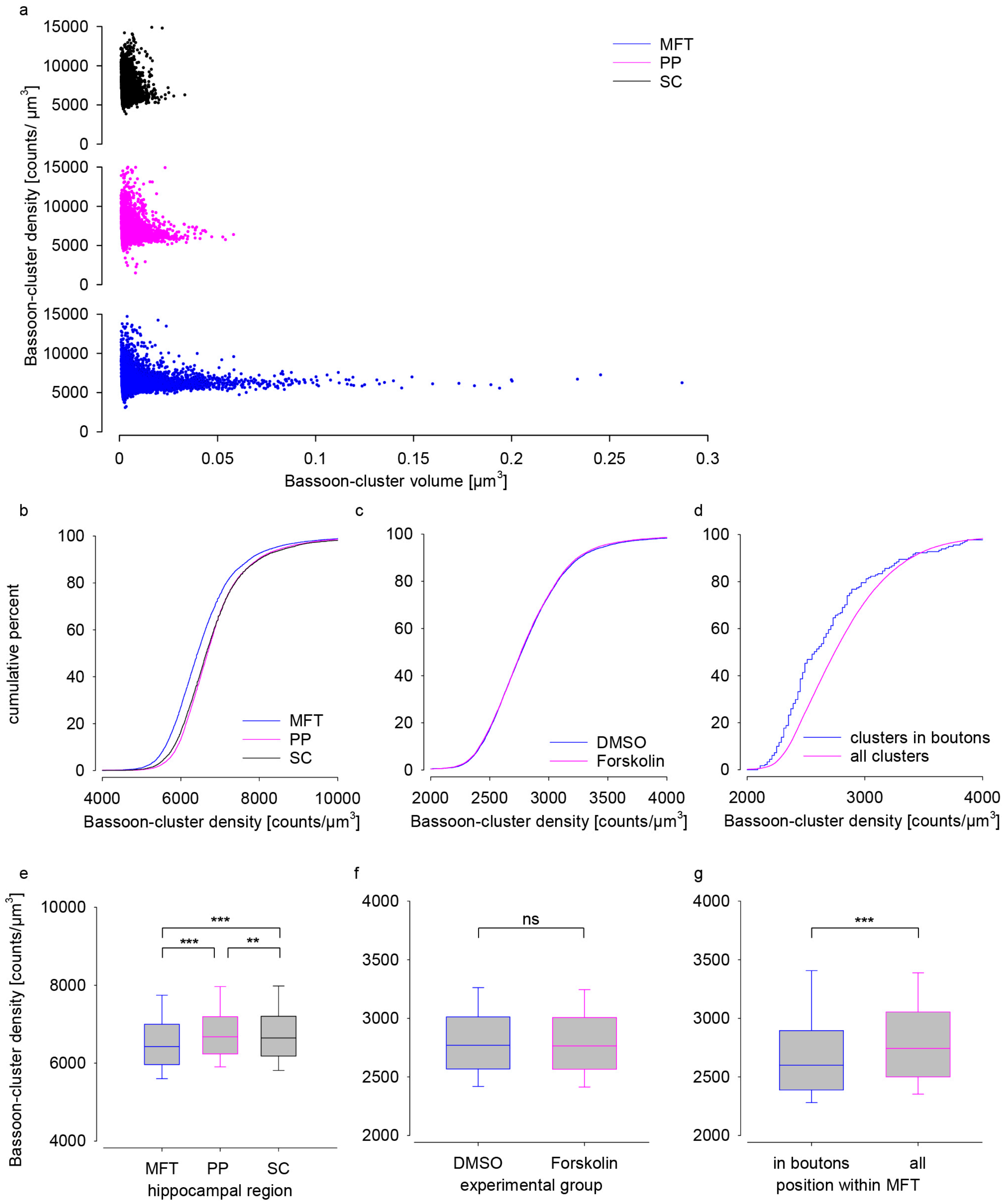


**a** Scatter plots of cluster density vs. volume in mossy fiber tract (MFT), perforant pathway (PP) and Schaffer collaterals (SC). **b-d:** Cumulative plots of cluster density in 3D recordings of **b** MFT (blue), PP (magenta) and SC (black), **c** in MFT after DMSO (blue) or Forskolin (magenta) treatment and **d** in MFT comparing densities of all clusters per image (magenta) to those in identified mossy fiber boutons (blue). **e-g** Corresponding data as boxplots. Asterisks (** < 0.01, *** < 0.001) denote statistical significance between the groups.

**Supplementary Fig. S6. Bassoon clusters in an *en bloc* scan of the hippocampal mossy fiber tract.**

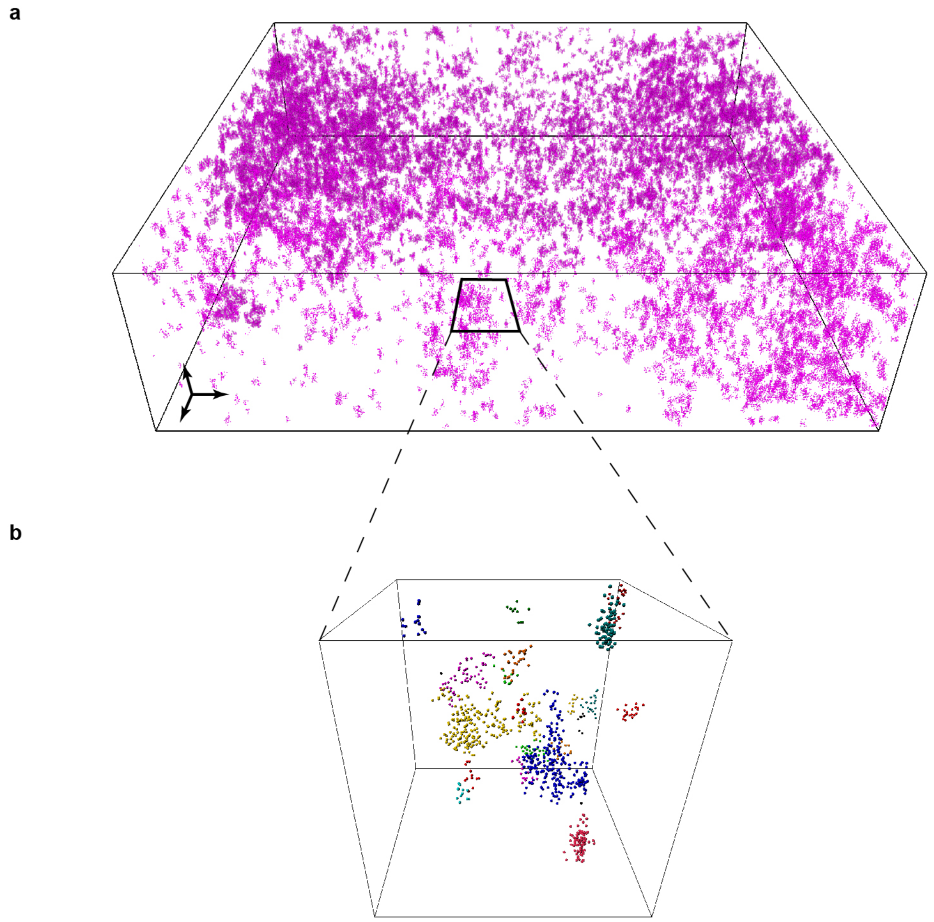


Bassoon signal in an entire *en bloc* image and a zoom-in of the marked box with color-coded individual clusters. Scale bars in the upper image = 2 µm x 2 µm x 2 µm (xyz) and box size below = 3 µm x 3 µm x 3 µm.

**Supplementary Fig. S7. Controls for antibody staining.**

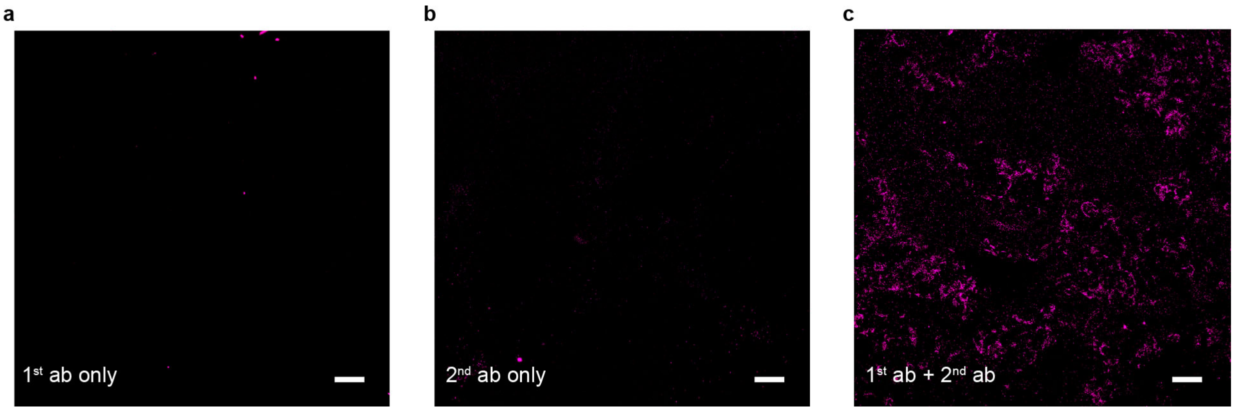


**a** Staining after omission of the Alexa647-goat anti-mouse Fab2 secondary antibody and **b** after omission of mouse anti-Bassoon monoclonal primary antibody. **c** Specific staining using both antibodies. Scale bar in all panels = 2 µm.

**Supplementary Fig. S8. Histogram of localization counts per Bassoon cluster in non-filtered data.**

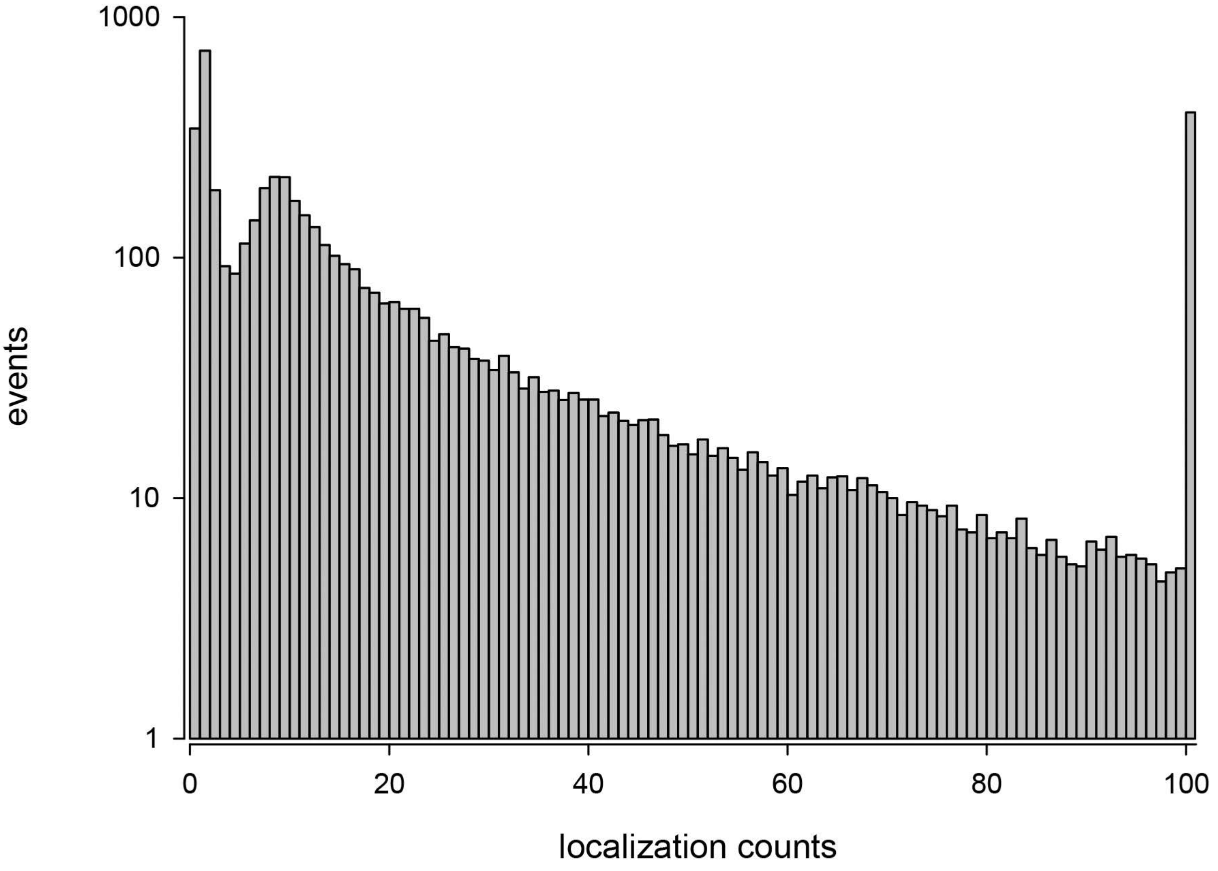


A low cut-off of 8 localizations was used for filtered data.

**Supplementary Table S1. Characteristics of Bassoon clusters in individual mossy fiber boutons.**

| bouton- number | bouton-volume [µm$^3$] | number of clusters | cluster volume median [µm$^3$] | cluster volume range [µm$^3$] |
|---|---|---|---|---|
| bouton 1 | 20.81 | 15 | 0.0067 | 0.0026-0.0128 |
| bouton 2 | 13.84 | 17 | 0.00877 | 0.0021-0.134 |
| bouton 3 | 13.56 | 9 | 0.0152 | 0.0023-0.0568 |
| bouton 4 | 7.94 | 11 | 0.0086 | 0.0026-0.0481 |
| bouton 5 | 8.67 | 4 | 0.0250 | 0.0020-0.0478 |
| bouton 6 | 3.28 | 1 | 0.0219 | 0 |
| bouton 7 | 2.88 | 1 | 0.0283 | 0 |
| bouton8 | 2.65 | 1 | 0.0041 | 0 |
| bouton 9 | 2.33 | 5 | 0.0204 | 0.0033-0.108 |
| bouton 10 | 24.34 | 33 | 0.0211 | 0.0019-0.177 |
| bouton 11 | 2.59 | 7 | 0.0177 | 0.0045-0.264 |
| bouton 12 | 2.69 | 2 | 0.0433 | 0.0354-0.0512 |
| bouton 13 | 4.30 | 8 | 0.110 | 0.0022-0.279 |
| bouton 14 | 3.18 | 7 | 0.0052 | 0.0004-0.0149 |
| bouton 15 | 1.33 | 1 | 0.0054 | 0 |
| bouton 16 | 3.85 | 4 | 0.0173 | 0.0069-0.112 |
| bouton 17 | 4.36 | 7 | 0.0406 | 0.0181-0.139 |
| bouton 18 | 2.91 | 1 | 0.0620 | 0 |
| bouton 19 | 3.79 | 1 | 0.105 | 0 |
| bouton 20 | 32.18 | 45 | 0.0134 | 0.0018-0.167 |
| bouton 21 | 3.08 | 1 | 0.0100 | 0 |

Mean bouton volume = 7.84 µm$^3$; mean number of clusters = 8.62, mean cluster volume = 0.033 µm$^3$.
