# Supplementary material for: Targeted volumetric single-molecule localization microscopy of defined presynaptic structures in brain sections
Source: Commun Biol. 2021 Mar 25;4:407. doi: 10.1038/s42003-021-01939-z (PMC7994795; doi:10.1038/s42003-021-01939-z)
Supplement: Supplementary file 2 — Description of Additional Supplementary Files [file 42003_2021_1939_MOESM2_ESM.pdf]

Description of Additional Supplementary Files

File name: Supplementary Video 1
Description: 2-color 3D dSTORM volumetric presentation of a single synaptic contact with Bassoon (green) and Homer 1 (magenta).

File name: Supplementary Video 2
Description: Sequential 3D dSTORM volumetric presentation of Bassoon clusters and mEGFP in mossy fiber tract of a Thy1-mEGFP (Lsi1) mouse. Bassoon clusters are color-coded according to cluster size from blue to red, mEGFP in axons and boutons in cyan.

File name: Supplementary Data 1
Description: The Source data underlying Fig. 2g-j: panel 2g: Bassoon cluster length of all animals, panel 2h: Individual Bassoon cluster length of individual animals, panel 2i: Bassoon cluster counts of all animals, panel 2j: Bassoon cluster counts of individual animals.

File name: Supplementary Data 2
Description: The Source data underlying Fig. 3g-h: panel 3g: Bassoon cluster volume in all clusters, panel 3h: Bassoon cluster volume in non-truncated clusters.

File name: Supplementary Data 3
Description: The Source data underlying Fig. 4e-j : panel 4e: Bassoon cluster counts in mossy fiber tract (MFT), perforant pathway (PP) and Schaffer collaterals (SC), panel 4f: Bassoon cluster lengths in MFT, PP and SC, panel 4g: Bassoon cluster volumes in MFT, PP and SC, panel 4h: Bassoon cluster counts in MFT, PP and SC, panel 4i: Bassoon cluster lengths in MFT, PP and SC, panel 4j: Bassoon cluster volumes in MFT, PP and SC.

File name: Supplementary Data 4
Description: The Source data underlying Fig. 5a-i: panel 5a: Bassoon cluster length in DMSO or Forskolin treated hippocampal slices of animal 1, panel 5b: Bassoon cluster counts in DMSO or Forskolin treated hippocampal slices of animal 1, panel 5c: Bassoon cluster volume in DMSO or Forskolin treated hippocampal slices of animal 1, panel 5d: Bassoon cluster length in DMSO or Forskolin treated hippocampal slices of animal 2, panel 5e: Bassoon cluster counts in DMSO or Forskolin treated hippocampal slices of animal 2, panel 5f: Bassoon cluster volume in DMSO or Forskolin treated hippocampal slices of animal 2, panel 5g: Bassoon cluster length in DMSO or Forskolin treated hippocampal slices of animal 3, panel 5h: Bassoon cluster counts in DMSO or Forskolin treated hippocampal slices of animal 3, panel 5i: Bassoon cluster volume in DMSO or Forskolin treated hippocampal slices of animal 3.

File name: Supplementary Data 5

Description: The source data underlying Fig. 6e-i: panel 6e: Bassoon cluster length for data in the histogram, panel 6f: Bassoon cluster length for data in box plots, panel 6g: Median values of Bassoon cluster length after data reduction, panel 6h: Bassoon cluster localization counts in all imaging conditions, panel 6i: Bassoon cluster length in all imaging conditions.

File name: Supplementary Data 6
Description: The source data underlying Fig. 7d-i: panel 7d: Bassoon cluster localization counts in clusters in boutons and in all clusters of the image, panel 7e: Bassoon cluster length in clusters in boutons and in all clusters of the image, panel 7f: Bassoon cluster volume in clusters in boutons and in all clusters of the image, panel 7g: Bassoon cluster localization counts in clusters in boutons and in all clusters of the panel 7h: Bassoon cluster length in clusters in boutons and in all clusters of the image, panel 7i: Bassoon cluster volume in clusters in boutons and in all clusters of the image.

File name: Supplementary Data 7
Description: The source data underlying Supplementary Fig. S1c, e-g,i-k: panel S1c: Peak-to-peak distance between Bassoon and Homer clusters in side view, panel S1e: Cluster length in Bassoon clusters in side view, panel S1f: Cluster width in Bassoon clusters in side view, panel S1g: Localization counts in Bassoon clusters in side view, panel S1i: Cluster length in Bassoon clusters in plane view, panel S1j: Cluster width in Bassoon clusters in plane view., panel S1k: Localization counts in Bassoon clusters in plane view.

File name: Supplementary Data 8
Description: The source data underlying Supplementary Fig. S3a-c: panel S3a: average localization intensities (AD counts) within the z-range of the image, panel S3b: average localization counts within the z-range of the image, panel S3c: localization intensity /local background within the z-range of the image.

File name: Supplementary Data 9
Description: The source data underlying Supplementary Fig. S5a-g: panel S5a: Bassoon cluster volumes and densities in in mossy fiber tract (MFT), perforant pathway (PP) and Schaffer collaterals (SC), panel S5b: Bassoon cluster densities in MFT PP and SC, panel S5c: Bassoon cluster densities in DMSO or Forskolin treated hippocampal slices, panel S5d: Bassoon cluster densities in clusters in boutons and in all clusters of the image, panel S5e: Bassoon cluster densities in MFT PP and SC, panel S5f: Bassoon cluster densities in DMSO or Forskolin treated hippocampal slices, panel S5g: Bassoon cluster densities in clusters in boutons and in all clusters of the image.

File name: Supplementary Data 10
Description: The source data underlying Supplementary Fig. S8: Bassoon cluster localization counts in unfiltered data.
