# Supplementary material for: Targeted volumetric single-molecule localization microscopy of defined presynaptic structures in brain sections
Source: Commun Biol. 2021 Mar 25;4:407. doi: 10.1038/s42003-021-01939-z (PMC7994795; doi:10.1038/s42003-021-01939-z)
Supplement: Supplementary file 15 — Reporting Summary [file 42003_2021_1939_MOESM15_ESM.pdf]

nature research

Corresponding author(s): Anna-Leena Sirén

Last updated by author(s): 02/24/2021

Reporting Summary

Nature Research wishes to improve the reproducibility of the work that we publish. This form provides structure for consistency and transparency in reporting. For further information on Nature Research policies, see our Editorial Policies and the Editorial Policy Checklist.

Statistics

For all statistical analyses, confirm that the following items are present in the figure legend, table legend, main text, or Methods section.

n/a	Confirmed	
☐	☒	The exact sample size (*n*) for each experimental group/condition, given as a discrete number and unit of measurement
☐	☒	A statement on whether measurements were taken from distinct samples or whether the same sample was measured repeatedly
☐	☒	The statistical test(s) used AND whether they are one- or two-sided<br>*Only common tests should be described solely by name; describe more complex techniques in the Methods section.*
☒	☐	A description of all covariates tested
☐	☒	A description of any assumptions or corrections, such as tests of normality and adjustment for multiple comparisons
☐	☒	A full description of the statistical parameters including central tendency (e.g. means) or other basic estimates (e.g. regression coefficient) AND variation (e.g. standard deviation) or associated estimates of uncertainty (e.g. confidence intervals)
☐	☒	For null hypothesis testing, the test statistic (e.g. *F*, *t*, *r*) with confidence intervals, effect sizes, degrees of freedom and *P* value noted<br>*Give P values as exact values whenever suitable.*
☒	☐	For Bayesian analysis, information on the choice of priors and Markov chain Monte Carlo settings
☒	☐	For hierarchical and complex designs, identification of the appropriate level for tests and full reporting of outcomes
☒	☐	Estimates of effect sizes (e.g. Cohen's *d*, Pearson's *r*), indicating how they were calculated

Our web collection on statistics for biologists contains articles on many of the points above.

Software and code

Policy information about availability of computer code

Data collection	Andor-Solis (Andor, ACAL BFI)
Data analysis	Rapid STORM 3.3.1., FIJI-ImageJ, Imaris-Bitplane 9.2., Python 2.7., Python3.5, Python 3.6, Sigmaplot 12 and 14 (Systat)

For manuscripts utilizing custom algorithms or software that are central to the research but not yet described in published literature, software must be made available to editors and reviewers. We strongly encourage code deposition in a community repository (e.g. GitHub). See the Nature Research guidelines for submitting code & software for further information.

Data

Policy information about availability of data

All manuscripts must include a data availability statement. This statement should provide the following information, where applicable:

- Accession codes, unique identifiers, or web links for publicly available datasets
- A list of figures that have associated raw data
- A description of any restrictions on data availability

The data that support the findings of this study including custom Python codes are available from the corresponding authors upon reasonable request.

Field-specific reporting

Life sciences study design

All studies must disclose on these points even when the disclosure is negative.

Sample size	We recorded in each measurement over 100 active zones in each image scan. The numbers of animals used in each measurement is given in the figure legend and in methods.
Data exclusions	No data were excluded.
Replication	Each measurement was done at least in duplicate at minimum for the number of samples/animal/group indicated in each figure legend.
Randomization	The brain slices in the forskolin-experiment were randomized for treatment (forskolin/DMSO). Otherwise randomization was not needed as we measured active zones in one strain of mice.
Blinding	The investigators were blinded for data analysis.

Reporting for specific materials, systems and methods

We require information from authors about some types of materials, experimental systems and methods used in many studies. Here, indicate whether each material, system or method listed is relevant to your study. If you are not sure if a list item applies to your research, read the appropriate section before selecting a response.

Materials & experimental systems

n/a	Involved in the study	
☐	☒	Antibodies
☒	☐	Eukaryotic cell lines
☒	☐	Palaeontology and archaeology
☐	☒	Animals and other organisms
☒	☐	Human research participants
☒	☐	Clinical data
☒	☐	Dual use research of concern

Methods

n/a	Involved in the study	
☒	☐	ChIP-seq
☒	☐	Flow cytometry
☒	☐	MRI-based neuroimaging

Antibodies

Antibodies used	the following antibodies were used:<br>mouse monoclonal anti-Bassoon (clone SAP7F407, cat# ADI-VAM-PS003-F, Enzo Life Sciences); rabbit polyclonal anti-Homer1 (Synaptic Systems cat# 16003), rabbit polyclonal and mAb anti-Zinc transporter 3 (ZnT3) (Synaptic Systems cat # 197 002 and cat #197 011), Chromotek GFP VHH recombinant binding protein (cat# gt250), Alexa 647 conjugated goat anti-mouse antigen binding fragment (Fab2, cat# A21237), Alexa 532 conjugated goat anti-mouse Fab (cat#A11002), Alexa 647 conjugated goat anti-rabbit Fab (cat#A21245), Alexa 532 conjugated goat anti-rabbit Fab (cat# A11009) all from Invitrogen/Thermo Fisher
Validation	Specificity of the primary antibodies is stated in detail in the manuscript (Results and data in Figure 2).

Animals and other organisms

Policy information about studies involving animals; ARRIVE guidelines recommended for reporting animal research

Laboratory animals	Brain sections from male 12 or 24 week old Thy1-EGFP(M) or male 12 week old Thy1-mEGFP (Lsi1) mice on C57/BL6 background were used in all experiments.
Wild animals	Study did not involve wild animals.
Field-collected samples	Study did not involve samples collected from the field.
Ethics oversight	Animal procedures were approved and performed in accordance with the guidelines of institutional and regulatory authorities (Permit number RUF-55.2.2-2532-2-572-16 of the district government of Lower Franconia), the EU Directive 2010/63/EU and the United States Public Health Service's Policy on Humane Care and Use of Laboratory Animals.

Note that full information on the approval of the study protocol must also be provided in the manuscript.
